# Blood Glucose Level Time Series Forecasting: Nested Deep Ensemble Learning Lag Fusion

**DOI:** 10.3390/bioengineering10040487

**Published:** 2023-04-19

**Authors:** Heydar Khadem, Hoda Nemat, Jackie Elliott, Mohammed Benaissa

**Affiliations:** 1 Department of Electronic and Electrical Engineering, University of Sheffield, Sheffield S10 2TN, UK; 2 Department of Oncology and Metabolism, University of Sheffield, Sheffield S10 2TN, UK; 3 Department of Diabetes and Endocrinology, Sheffield Teaching Hospitals, Sheffield S5 7AU, UK

**Keywords:** deep learning, time-series forecasting, blood glucose, diabetes, ensemble learning, artificial neural network

## Abstract

Blood glucose level prediction is a critical aspect of diabetes management. It enables individuals to make informed decisions about their insulin dosing, diet, and physical activity. This, in turn, improves their quality of life and reduces the risk of chronic and acute complications. One conundrum in developing time-series forecasting models for blood glucose level prediction is to determine an appropriate length for look-back windows. On the one hand, studying short histories foists the risk of information incompletion. On the other hand, analysing long histories might induce information redundancy due to the data shift phenomenon. Additionally, optimal lag lengths are inconsistent across individuals because of the domain shift occurrence. Therefore, in bespoke analysis, either optimal lag values should be found for each individual separately or a globally suboptimal lag value should be used for all. The former approach degenerates the analysis’s congruency and imposes extra perplexity. With the latter, the fine-tunned lag is not necessarily the optimum option for all individuals. To cope with this challenge, this work suggests an interconnected lag fusion framework based on nested meta-learning analysis that improves the accuracy and precision of predictions for personalised blood glucose level forecasting. The proposed framework is leveraged to generate blood glucose prediction models for patients with type 1 diabetes by scrutinising two well-established publicly available Ohio type 1 diabetes datasets. The models developed undergo vigorous evaluation and statistical analysis from mathematical and clinical perspectives. The results achieved underpin the efficacy of the proposed method in blood glucose level time-series prediction analysis.

## 1. Introduction

Type 1 diabetes is a chronic metabolic disorder [1]. The disease is currently incurable [2,3]. Nevertheless, its effective management can dramatically mitigate the symptoms and the risk of associated short-term and long-term complications [4,5]. Accordingly, people with type 1 diabetes and their potential carers are normally educated on the standard practices to control the illness [6,7,8].

Self-management of type 1 diabetes is, however, burdensome and prone to human errors [9,10,11]. Hence, automating the management tasks would be highly beneficial [12,13]. Some developments have already been made related to this concern [14,15,16]. For example, technological breakthroughs, such as continuous glucose monitoring biosensors [17,18] and insulin pumps [19,20], nowadays, serve myriads of type 1 diabetes patients. The former, in a minimally invasive fashion, takes regular snapshots of blood glucose levels in alignment with the general advice on a frequent review of glycaemic state [21,22]. The latter semiautomates insulin administration, requiring minimum user interference [23,24,25]. Moreover, there are ongoing efforts to develop fully noninvasive continuous blood glucose level monitoring sensors to help more effective diabetes management [26,27,28,29].

Despite the advancements achieved so far, continued progress in the automation process is still demanded to further facilitate and effectuate the management of type 1 diabetes [30,31]. In this respect, engineering accurate blood glucose predictor devices would be game changing [32,33]. Such instruments can provide early warning about possible adverse glycaemic events so that automated or nonautomated pre-emptive measures can be taken [34,35]. Additionally, these devices are a prerequisite for the advent of a closed-loop artificial pancreas as the current vision for the ultimate automated management of type 1 diabetes [36,37].

For predicting blood glucose levels, physiological, data-driven, and hybrid modelling approaches can be pursued [38,39]. In the data-driven approach, also used in this research, current and past values of diabetes-management-related variables are studied to project future blood glucose excursion [38,40].

For constructing data-driven blood glucose level predictors, one of the three main categories of time-series forecasting approaches is typically used: classical time-series forecasting, traditional machine learning, or deep learning analysis. Among these, deep learning, as a member of the modern artificial intelligence family, has proven potency in solving complicated computational tasks, including complex time-series forecasting [41,42,43,44,45,46].

Predicting the blood glucose levels of individuals with type 1 diabetes is a convoluted forecasting mission due to the highly erratic behaviour of the phenomenon [47]. Thus, in line with many other time-series forecasting areas, deep learning has gained enormous popularity in the blood glucose level prediction realm [48,49]. Subsequently, extensive research has been underway to advance the analysis. Notwithstanding all the enhancements in this field so far, there still exist challenges to be addressed adequately [50]. This work contributes to addressing one such challenge.

When applying deep learning algorithms for data-driven time-series blood glucose level forecasting, lag observations of data are studied to predict specific future values. Here, a quandary is to select the appropriate length of history to be investigated. This issue is even more pronounced when considering the fact that due to the significant discrepancy in the blood glucose profile across type 1 diabetes patients, the common practice is to generate personalised models. In this circumstance, finding an optimal length of history separately for each individual entails further disparity and complexity in the analysis. To address this difficulty, the present work suggests a compound lag fusion approach by exploiting the potential of nested ensemble learning over typical ensemble learning analysis. This is the first paper, to the best of our knowledge, that incorporates nested meta-learning analysis in the field of blood glucose level prediction.

The rest of the article is outlined as follows. Section 2 reviews some recent studies on type 1 diabetes blood glucose level prediction. Section 3 concisely describes the datasets used in this research. Section 4 explains model development and assessment analysis. Section 5 presents the results of the model assessment analysis along with the relevant discussions. Finally, Section 6 summarises and concludes the work.

## 2. Literature Survey

In the following, a number of recent articles on data-driven blood glucose level prediction are succinctly overviewed. For further alignment with the contents of this study, the focus of this overview is on the application of state-of-the-art machine learning techniques and the use of Ohio type 1 diabetes datasets for model development and evaluation. A more comprehensive review of the latest revolutions in the blood glucose level prediction area can be studied at these references [51,52,53,54].

A recent article offered a multitask approach for blood glucose level prediction by experimenting on the Ohio datasets [55]. The methods are based on the concept of transfer learning. The study explicitly targets addressing the challenge of the need for extensively large amounts of data for personalised blood glucose level prediction. For this purpose, it suggests pre-training a model on a source domain and a multitask model on the whole dataset and then using these learning experiences in constructing personalised models. The authors showcase the efficacy of their propositions by comparing the performance of their approach with sequential transfer learning and subject-isolated learning.

An autonomous channel setup was recently presented for deep learning blood glucose level prediction using the Ohio datasets [56]. The proposed method chose the history lengths for different variables adaptively by affecting the time-dependency scale. The crux is to avoid dismissing useful information from variables with enduring influence and engaging uninformative data from variables with transient impact at the same time. The models generated in the study undergo comparison analysis with standard non-autonomous channel structures deploying mathematical and clinical assessments.

A deep learning approach based on dilated recurrent neural networks accompanied by transfer learning concepts is introduced for blood glucose level prediction [57]. In the study, personalised models are created for individuals with type 1 diabetes using an Ohio dataset. The method is examined for short-term forecasting tasks. Its supremacy over standard methods, including autoregressive models, support vector regression, and conventional neural networks, is shown.

Another study suggests an efficient method for univariate blood glucose level prediction [58]. In the analysis, recurrent neural networks were used as learners. The learners are trained in an end-to-end approach to predict future blood glucose levels 30 and 60 min in advance using only histories of blood glucose data. The models are developed and assessed using an Ohio dataset. The results achieved are comparable with the state-of-the-art research on the dataset. In addition to accuracy analysis, the study investigates the certainty in the predictions. To do so, a parameterised univariate Gaussian is tasked with calculating the standard deviation of the predictions as a representative of uncertainty.

Employing the concepts of the Internet of things, a study compares four broadly used models of glycaemia, including support vector machine, Bayesian regularised neural network, multilayer perceptron, and Gaussian approach [59]. These models are used to investigate the possibility of completing the data collected from 25 individuals with type 1 diabetes by mapping intricate patterns of data. The findings highlight the potential of such analysis in contributing to improved diabetes management. Further, among the approaches examined, Bayesian regularised neural networks outperform others by delivering the best root mean square error and coefficient of determination.

## 3. Material

For generating blood glucose level prediction models, this study uses two well-established, publicly accessible Ohio type 1 diabetes datasets [60]. The first dataset includes data for six individuals with type 1 diabetes. The participants’ age at the time of data collection was in a range of 40 to 60 years. The sample comprised four females and two males. This dataset was initially released for the first blood glucose challenge in Knowledge Discovery at the Healthcare Data conference in 2018. This dataset is referred to as the Ohio 2018 dataset hereafter. The second dataset also contains six people with type 1 diabetes, different from those in the first dataset. The data contributors in this dataset were in an age range of 20 to 80 years at the point of data acquisition. Five of them were male and one female. This dataset was originally distributed for the second blood glucose level prediction challenge in Knowledge Discovery at the Healthcare Data conference in 2020. Hereafter, we refer to this dataset as the Ohio 2020 dataset.

Both datasets contain diabetes-related modalities, including blood glucose, physical activity, carbohydrate intake, and bolus insulin injection. Blood glucose and bolus insulin data were collected automatically using physiological sensors. For the former, a Medtronic Enlite continuous glucose monitoring device was used. For the latter, patients in the Ohio 2018 dataset wore a Basis Peak fitness band that collected heart rate data as a representative of physical activity. Alternatively, subjects in the Ohio 2020 dataset wore an Empatica Embrace fitness band that tracked the magnitude of acceleration as a representative of physical activity data. On the other hand, carbohydrate and bolus insulin data were self-reported by individuals in both datasets.

In both datasets, data were collected for eight weeks. The data come with the training and testing set already separated by the data collection and distribution team. The last ten days of data are allocated as a testing set and the remaining former data points as the training set. In the present study, using training sets only, bespoke predictive models are created for future values of blood glucose levels from historical values of blood glucose itself as the indigenous variable, along with exogenous variables of physical activity, carbohydrate intake, and bolus insulin injection. The testing sets are then used to evaluate the generated models. Table 1 displays individuals’ identification number, sex, and age information together with a short representation of the statistical properties of blood glucose as the intrinsic variable in the dataset. A more comprehensive description of the Ohio datasets and the data collection process can be found in the original documentation [60].

## 4. Methods

This section explicates the methodological implementations for blood glucose level prediction model generation and evaluation. First, some curation steps performed to prepare the data for formal prediction modelling analysis are explained. Next, time-series forecasting models constructed for blood glucose level prediction are described. After that, the criteria considered for evaluating the generated predictive models are presented. Finally, statistical analysis operated on the model outputs is outlined.

### 4.1. Data Curation

The following pre-modelling curation steps are operated on the raw data to render the ensuing formal deep learning prediction modelling analysis more effective.

#### 4.1.1. Missingness Treatment

The first data curation stage deals with the missing values presented in the automatically collected blood glucose and physical activity data. At the beginning and end of the blood glucose and physical activity series, there are some timespans where data are absent. This unavailability occurred because the subject did not start and finish wearing the sensing devices exactly at the same time. As an initial missing value treatment step, the head and tail of all series are trimmed by removing the void timestamps so that variables start and end from the same point. Afterwards, the linear interpolation technique is used to fill in missing values in the training sets of blood glucose and physical activity. Alternatively, for the testing sets of these modalities, the linear extrapolation technique is used to fill in missing values. This technique precludes future value observation in the evaluation stage, so the models created possess applicability for real-time monitoring.

#### 4.1.2. Sparsity Handling

The sparsity of the self-reported carbohydrate and bolus insulin data is the next pre-modelling issue to be addressed. A reasonable assumption as to the unavailable values of these modalities in the majority of timestamps is that there has been no occurrence to be reported in those points. Therefore, for these two modalities, as a simple yet acceptable practice, zero values are assigned to non-reported timestamps.

#### 4.1.3. Data Alignment

Another data curation step is to unify the frequency of exogenous modalities and align their timestamps with the blood glucose level as the indigenous variable. Initially, acceleration data are downsampled from a one-minute frequency to a five-minute frequency. For this purpose, the entries in the nearest neighbourhood to blood glucose timestamps are kept, and the remaining data points are removed. Following that, timestamps of all extrinsic variables are aligned with those of blood glucose levels with the minimum possible shifts.

#### 4.1.4. Data Transformation

As the next data curation step, as a common practice, feature values are converted into a standardised form that machine learning models can analyse more effectively. For each variable, first, the average of training set values is subtracted from all values in both the training and testing sets. Then, all obtained values are divided by the standard deviation of the training set to make unit variance variables.

#### 4.1.5. Stationarity Inspection

Stationary time-series data have statistical characteristics, including variance and mean, that do not change over time. In this data treatment step, the stationarity condition in the time-series data is satisfied. By conducting the feature transformation step explained in Section 4.1.4, the variances in the series are stabilised. To stabilise the mean of the series, the first-order differencing method is applied. Subsequently, the outcomes are examined using two prevalent statistical tests of Kwiatkowski–Phillips–Schmidt–Shin [61] and Augmented Dickey–Fuller [62], where both confirm the stationary of the series.

#### 4.1.6. Problem Reframing

The final data curation phase translates the time-series blood glucose level prediction question to the supervised machine learning language. Hence, pairs of independent and dependent variables need to be constructed from the time-series data. To this end, a rolling window approach is used to appoint sequences of lag observations for blood glucose, physical activity, carbohydrate, and bolus insulin as the independent variables and sequences of blood glucose in the prediction horizon as the dependent variable.

### 4.2. Modelling

This subsection describes time-series forecasting models created for blood glucose level prediction 30 and 60 min into the future. This work undertakes a sequence-to-sequence fashion for multi-step-ahead time-series prediction. Prior to explaining the formal modelling process, it is useful to provide a brief explanation of stacking as an ensemble learning variation used in this work.

#### 4.2.1. Preliminary

Ensemble learning is an advanced machine learning method that attempts to improve analysis performance by combining the decisions of multiple models [63]. Stacking is a type of ensemble learning in which a meta-learner intakes predictions of a number of base learners as an input feature to make final decisions [64].

#### 4.2.2. Model Development

**Figure 1:**
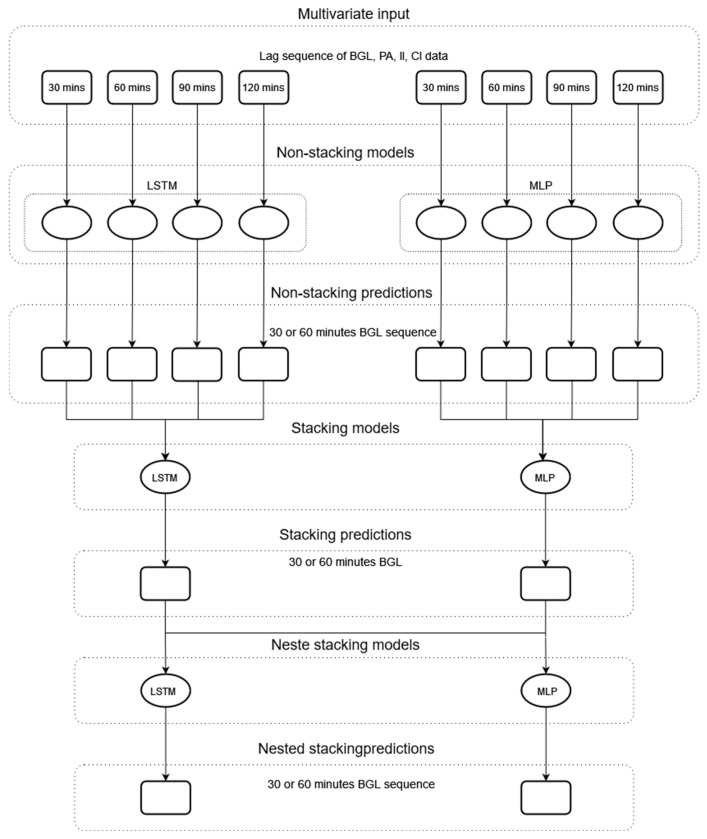
Blueprint for generating non-stacking, stacking, and nested stacking blood glucose level prediction models. Rectangular and oval blocks represent sequences of lag or future data and regression learners, respectively. Note. BGL: blood glucose level; PA: physical activity; II: insulin injection; CI: carbohydrate intake; LSTM: long short-term memory; MLP: multilayer perceptron.

The diagram in Figure 1 displays the procedure contrived in this work for model creation. According to the diagram, the models are constructed by training three categories of learners: non-stacking, stacking, and nested stacking. The models generated based on the block diagram in Figure 1 are described below.

A non-stacking model takes a specific length of historical blood glucose, physical activity, carbohydrate, and bolus insulin data as multivariate input and returns a sequence of forecasted future blood glucose levels over a predefined prediction horizon of 30 or 60 min. According to the diagram in Figure 1, for each prediction horizon of 30 and 60 min, eight non-stacking models are created in aggregate. For this purpose, a multilayer perceptron network and a long short-term memory network are trained separately on four different lag lengths of 30, 60, 90, and 120 min.

A stacking model is a meta-model that takes sequence predictions from four non-stacking models with a homogenous learner (multilayer perceptron network or long short-term memory network) as multivariate input and fuses them to generate new prediction outputs. According to v, for each prediction horizon of 30 and 60 min, two stacking models are created, one with multilayer perceptron networks and the other with long short-term memory networks as the underlying embedded learners.

A nested stacking model is a nested meta-model. It receives the outcomes of the two stacking models described above as multivariate inputs and returns new predictions. As can be seen in Figure 1, two nested stacking models are generated for each prediction horizon of 30 and 60 min; one employs a multilayer perceptron network and the other a long short-term memory network as the nested stacking learner.

According to Figure 1, in all model creation scenarios, the learners recruited are either multilayer perceptron or long short-term memory networks. For simplicity and coherency, all multilayer perceptron networks have similar architectures consisting of an input layer, a hidden dense layer with 100 nodes, followed by another dense layer as output. Additionally, all long short-term memory networks are the vanilla type with an input layer, a hidden 100-node LSTM layer, and a dense output layer. Given the five-minute resolution of time-series data investigated, the number of nodes in the output layer is 6 and 12 for 30 min and 60 min prediction horizons, respectively. In all networks, He uniform is set as the initialiser, Adam as the optimiser, ReLU as the activation function, and mean square error as the loss function. Moreover, in all training scenarios, epoch size and batch size are set to 100 and 32, respectively. In addition, the learning rate is initiated from 0.01, and then using the ReduceLROnPlateau callback, it is reduced by a factor of 0.1 once the validation loss reduction stagnates with patience of ten iterations.

### 4.3. Model Assessment

This section describes the analyses performed to validate the functionality of the developed blood glucose level prediction models. The generated models are assessed from regression, clinical, and statistical perspectives, as discussed below. 

#### 4.3.1. Regression Evaluation

Four broadly applied regression metrics are determined to verify the performance of the constructed models from a mathematical viewpoint. Mean absolute error (Equation (1)), root mean square error (Equation (2)), and mean absolute percentage error (Equation (3)) rate the accuracy of predictions. Further, the coefficient of determination (Equation (4)) measures the correlation between the reference and predicted blood glucose levels.

(1) $$MAE=\left(\sum_{i=1}^{N}\left|{BGL}_{i}-{B\hat{G}L}_{i}\right|\right)/N$$

(2) $$RMSE=\sqrt{(\sum_{i=1}^{N}\left({BGL}_{i}-{B\hat{G}L}_{i}{)}^{2}\right)/N}$$

(3) $$MAPE=((\sum_{i=1}^{N}\left|\left({BGL}_{i}-{B\hat{G}L}_{i}\right)/{BGL}_{i}\right|)/N)\times 100$$

(4) $$r^{2}=1-((\sum_{i=1}^{N}{\left({BGL}_{i}-{B\hat{G}L}_{i}\right)}^{2})(\sum_{i=1}^{N}{\left({BGL}_{i}-\bar{BGL}\right)}^{2}))$$

where MAE: mean absolute error; BGL: blood glucose level; N: the size of the testing set; RMSE: root mean square error; MAPE: mean absolute prediction error; r^2^: coefficient of determination.

#### 4.3.2. Clinical Evaluation

Two criteria are employed to evaluate the developed models from a clinical standpoint. One criterion is the Matthew’s correlation coefficient [65]. It is a factor fundamentally used for assessing the effectuality of binary classifications. In this work, this metric, calculated as Equation (5), is exploited to investigate the potency of the blood glucose prediction models in discriminating adverse glycaemic events from euglycaemic events. Hereby, an adverse glycaemic event is defined as a blood glucose level lower than 70 mg/dL (hypoglycaemia) or more than 180 mg/dL (hyperglycaemia), and a euglycaemia event as a blood glucose level between 70 mg/dL and 180 mg/dL.

(5) $$MCC=\left(TP\times TN-FP\times FN\right)/\sqrt{\left(TP+FP\right)\left(TP+FN\right)\left(TN+FP\right)\left(TN+FN\right)}$$

where TP: true positive (the count of correctly predicted adverse glycaemic events); TN: true negative (the count of correctly predicted euglycaemic events); FP: false positive (the count of falsely predicted adverse glycaemic events); FN: false negative (the count of falsely predicted euglycaemic events).

The other considered clinical evaluation criterion is surveillance error [66]. It is based on error grid analysis to identify the clinical risk of inaccuracies in blood glucose level predictions. Detailed calculations of surveillance error can be found in the original article [66]. However, a concise elucidation of the outcome of the calculations is as follows. A unitless error value is measured for each predicted blood glucose level. Errors smaller than 0.5 indicate clinically risk-free predictions. Errors between 0.5 and 1.5 indicate clinically slight-risk predictions. Errors between 1.5 and 2.5 indicate clinically moderate-risk predictions. Errors between 2.5 and 3.5 indicate clinically high-risk predictions. Finally, errors bigger than 3.5 indicate clinically critical-risk predictions. We adopt two evaluation metrics based on surveillance error calculation outcomes. One is the average of surveillance errors across the entire testing set, and the other is the proportion of obtained surveillance errors less than 0.5 (clinically riskless predictions) across the entire testing set.

#### 4.3.3. Statistical Analysis

Statistical analysis is conducted for further side-by-side performance assessment for different models. In this sense, the non-parametric Friedman test is exercised to compare the outcomes of different models [67]. This test is privileged for inter-model comparative analysis across multiple datasets with no normality assumption requirement as opposed to the counterpart ANOVA test [68]. In this study, the test is assigned to compare the performance of different types of models considering individuals as independent data sources. To do so, a significant level of five percent is considered to examine the consistency of results achieved for evaluation metrics. The null hypothesis for the test is that the results of the non-stacking, stacking, and nested stacking models have identical distributions. In the next step, for cases where the global Friedman test detects the existence of a statistically significant difference amongst the models’ performance, the local Nemenyi test [69], as a post hoc procedure, compares the models in a pairwise manner. In this multi-comparison analysis, the Holm–Bonferroni method is used to adjust the significance level [70]. Finally, the heuristic critical difference approach is employed to visualise the outcomes of the post hoc analysis [71]. The statistical tests are operated on all evaluation metrics in both prediction horizons of 30 and 60 min. Both multilayer perceptron and long short-term memory networks are examined as learners separately.

## 5. Results and Discussion

This section presents the outcomes of model assessment analyses and the relevant discussion. Initially, the results of regression-wise and clinical-wise evaluation investigations are given for the non-stacking, stacking, and nested stacking models. Therein, for each metric, mean and standard deviation values achieved over five model runs are reported, a common practice in deep learning to counteract the stochastic nature of the analysis. After presenting the evaluation results, the results of the statistical analysis performed for more detailed comparison inspections between different types of models are exhibited.

The full evaluation results of the non-stacking models are compartmentalised in four tables given in Appendix A. Table A1 is dedicated to models with multilayer perceptron learners created on the Ohio 2018 dataset, Table A2 to models with multilayer perceptron learners created on the Ohio 2020 dataset, Table A3 to models with long short-term memory learners created on the Ohio 2018 dataset, and Table A4 to models with long short-term memory learners created on the Ohio 2020. 

In the non-stacking analysis, there are four modelling scenarios for each patient: blood glucose level prediction 30 and 60 min in advance, once assigning multilayer perceptron and once long short-term memory as the learner. As can be seen in the Appendix A tables, for each scenario, four models are created by training the learner on 30, 60, 90, or 120 min of historical data separately. Additionally, there are four parallel modelling scenarios for stacking and nested stacking analysis: blood glucose level prediction 30 and 60 min in advance, once employing multilayer perceptron and once long short-term memory as the last-level learner. On the other hand, one model is created for each scenario in stacking and nested stacking analysis because different lags are not separately studied.

To compare the stacking and nested stacking analyses with the non-stacking analyses, initially, for each patient, one of the four non-stacking models created for each modelling scenario is selected as the representative. Then, the representative non-stacking models are studied in parallel with the counterpart stacking and nested stacking models. To select the representative non-stacking models, first, the best evaluation metrics achieved in each modelling scenario are marked in bold font in the Appendix A tables. Subsequently, the model delivering the highest number of best-obtained evaluation metrics, highlighted in grey in the tables, is deemed as the representative. For eligibility, the results for these models are given in Table 2. Moreover, the complete evaluation results for the stacking and nested stacking models are recorded in Table 3 and Table 4 respectively.

**Figure 2:**
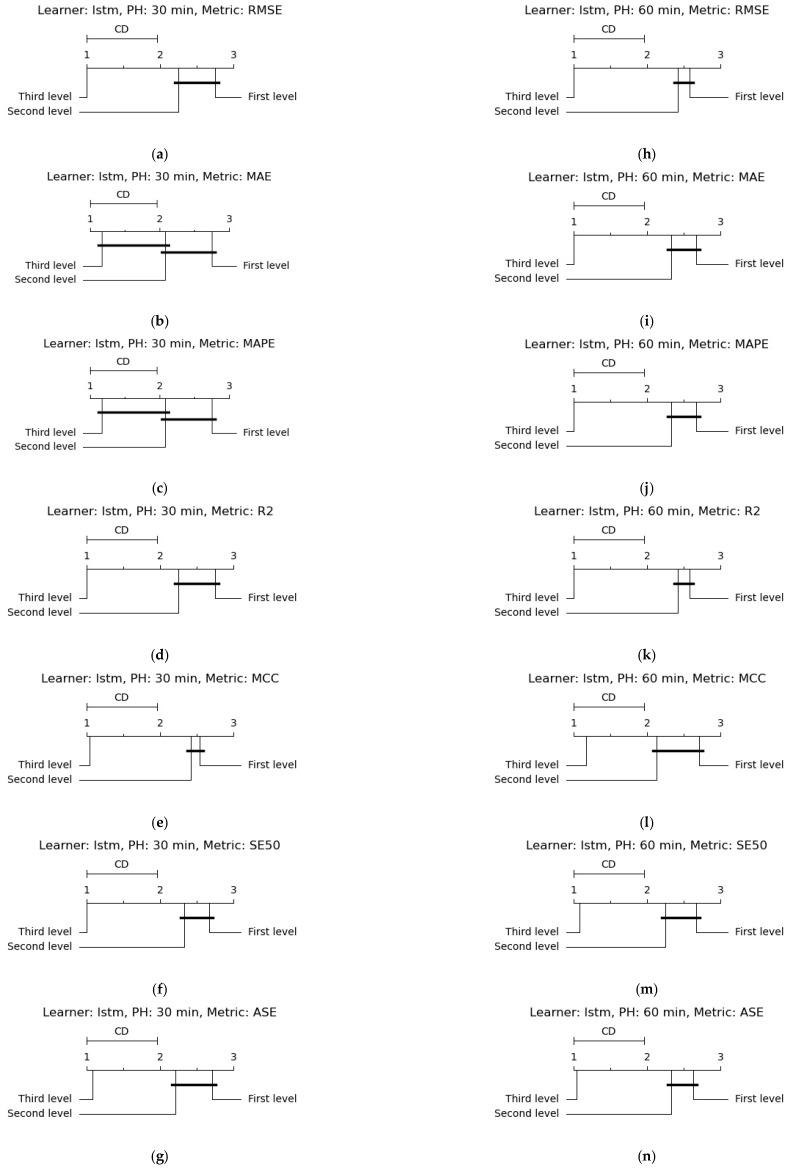
Critical difference diagrams based on Nemenyi test for pairwise comparison of the non-stacking, stacking, and nested stacking modelling approaches: (**a**) LSTM learner, 30 min PH, and RMSE metric, (**b**) LSTM learner, 30 min PH, and MAE metric, (**c**) LSTM learner, 30 min PH, and MAPE metric, (**d**) LSTM learner, 30 min PH, and r^2^ metric, (**e**) LSTM learner, 30 min PH, and MCC metric, (**f**) LSTM learner, 30 min PH, and SE50 metric, (**g**) LSTM learner, 30 min PH, and ASE metric, (**h**) LSTM learner, 60 min PH, and RMSE metric, (**i**) LSTM learner, 60 min PH, and MAE metric, (**j**) LSTM learner, 60 min PH, and MAPE metric, (**k**) LSTM learner, 60 min PH, and r^2^ metric, (**l**) LSTM learner, 60 min PH, and MCC metric, (**m**) LSTM learner, 60 min PH, and SE50 metric, (**n**) LSTM learner, 60 min PH, and ASE metric. Note. LSTM: long short-term memory; PH: prediction horizon; RMSE: root mean square error; MAE: mean absolute error; MAPE: mean absolute percentage error; r^2^: coefficient of determination; MCC: Matthew’s correlation coefficient; SE: surveillance error; ASE: average surveillance error.

After picking the representative non-stacking models, the overall performance of these models is compared with the stacking and nested stacking counterparts. To this end, first, the Friedman test is conducted on these models’ outcomes. *p*-values less than a significance level of 5% reveal scenarios in which there is a statistically meaningful distinction in the outputs of the three types of models for a specific evaluation metric. To elicit the performance difference for these cases, critical difference analysis integrated with post hoc Nemenyi test is used. The results of the critical difference analysis are shown in Figure 2. These diagrams show the average ranking of the modelling approaches in generating superior outcomes for a given evaluation metric. In each figure, models with statistically different average rankings are linked via a thick horizontal line. From Figure 2, the nested stacking models yielded superior evaluation outcomes overall. These findings substantiate the effectiveness of the propositions in addressing the challenge of lag optimisation while conducting enhanced outcomes. 

It is noteworthy that, according to the highlighted models in the Appendix A tables, an inconsistency in the efficient lag to be investigated for different patients, prediction horizons, and learners can be observed. In detail, the optimal lag is 30 min in 19 cases, 60 min in 19 cases, 90 min in 5 cases, and 120 min in 5 cases. Such disparity further accentuates the utility of the nested stacking analyses that efficaciously circumvent the lag optimisation process.

## 6. Summary and Conclusions

This work offers a nested meta-learning lag fusion approach to address the challenge of history length optimisation in personalised blood glucose level prediction. For this purpose, in lieu of examining different lengths of history from a search space and picking a local optimum for each subject or a global suboptimum for all subjects, all the lags in the search space are studied autonomously, and the results are amalgamated. A multilayer perceptron and long short-term memory network are initially trained on four different lags separately, resulting in four non-stacking models from each network. The outcomes of the four non-stacking multilayer perceptron models are then combined into new outcomes using a stacking multilayer perceptron model. Similarly, a stacking long short-term memory model fuses the results of the four non-stacking long short-term memory models. Finally, the decisions of the two stacking prediction models are ensembled once using a multilayer perceptron and once using a long short-term memory network as a nested stacking model. These investigations are performed for two commonly studied prediction horizons of 30 and 60 min in blood glucose level prediction research. The generated models undergo in-depth regression-wise, clinical-wise, and statistic-wise assessments. The results obtained substantiate the effectiveness of the proposed stacking and nested stacking methods in addressing the challenge of lag optimisation in blood glucose level prediction analysis.

## 7. Software and Code

For developing and evaluating blood glucose level prediction models, this research used Python 3.6 [72] programming. The libraries and packages employed include TensorFlow [73], Keras [73], Pandas [74], NumPy [75], Sklearn [76], SciPy [77], statsmodels [78], scikit-post hocs [79], and cd-diagram [80]. The source code for implementations is available on this Gitlab repository.

## Figures and Tables

**Table 1:** Demographic information of contributors and summary of statistical properties of blood glucose data (the focal modality) in the Ohio datasets.

Dataset	PID	Sex	Age	Set	Blood Glucose Data							
					Count	Range (mg/dL)	Mean (mg/dL)	SD (mg/dL)	MR (%)	HOR (%)	ER (%)	HRR (%)
2018	559	female	40–60	Train	10,655	40–400	167.53	70.44	12.06	3.65	55.98	40.37
				Test	2444	45–400	168.93	67.78	14.81	3.03	59.86	37.11
	563	male	40–60	Train	11,013	40–400	146.94	50.51	8.80	2.82	72.81	24.36
				Test	2569	62–313	167.38	46.15	4.71	0.70	60.45	38.85
	570	male	40–60	Train	10,981	46–377	187.5	62.33	5.73	1.97	42.97	55.07
				Test	2672	60–388	215.71	66.99	5.05	0.41	29.04	70.55
	575	female	40–60	Train	11,865	40–400	141.77	60.27	10.43	8.71	68.62	22.66
				Test	2589	40–342	150.49	60.53	4.94	5.37	63.50	31.13
	588	female	40–60	Train	12,639	40–400	164.99	50.51	3.69	1.04	63.56	35.40
				Test	2606	66–354	175.98	48.66	3.42	0.15	53.26	46.58
	591	female	40–60	Train	10,846	40–397	156.01	58.03	17.59	3.94	63.97	32.09
				Test	2759	43–291	144.83	51.42	3.15	5.18	67.27	27.55
2020	540	male	20–40	Train	11,914	40–369	136.78	54.75	9.76	7.08	72.66	20.25
				Test	2360	52–400	149.94	66.46	6.74	5.64	68.18	26.19
	544	male	40–60	Train	10,533	48–400	165.12	60.08	19.11	1.47	63.78	34.75
				Test	2715	62–335	156.48	54.14	15.47	1.22	68.29	30.50
	552	male	20–40	Train	8661	45–345	146.88	54.63	22.30	3.89	72.05	24.06
				Test	1792	47–305	138.11	50.23	85.71	3.57	80.02	16.41
	567	female	20–40	Train	10,750	40–400	154.43	60.88	24.91	6.75	63.40	29.84
				Test	2388	40–351	146.25	55.00	20.18	8.33	67.38	24.29
	584	male	40–60	Train	12,027	40–400	192.34	65.29	9.13	0.80	47.69	51.51
				Test	2661	41–400	170.48	60.76	12.40	1.01	61.86	37.13
	596	male	60–80	Train	10,858	40–367	147.17	49.34	25.35	2.08	73.99	23.93
				Test	2663	49–305	146.98	50.79	9.76	2.78	75.07	22.16

Note. PID: patient identification; SD: standard deviation; MR: missingness rate; HOR: hypoglycaemic rate; ER: euglycaemic rate; HRR: hyperglycaemic rate. Hypoglycaemia, euglycaemia, and hyperglycaemia refer to when the blood glucose level is, respectively, less than 70 mg/dL, between 70 and 180 mg/dL, and more than 180 mg/dL. Both hypoglycaemia and hyperglycaemia are adverse glycaemic events.

**Table 2:** The evaluation results for the best non-stacking models created using Ohio datasets.

Dataset	PID	Learner	PH	Evaluation Metric						
				RMSE ± SD (mg/dL)	MAE ± SD (mg/dL)	MAPE ± SD (%)	r^2^ ± SD (%)	MCC ± SD (%)	SE < 0.5 ± SD (%)	ASE ± SD
2018	559	MLP	30	19.65 ± 0.06	13.56 ± 0.03	8.78 ± 0.03	90.75 ± 0.05	0.77 ± 0.00	0.90 ± 0.00	0.19 ± 0.00
			60	31.36 ± 0.06	22.78 ± 0.06	15.18 ± 0.07	76.30 ± 0.08	0.63 ± 0.00	0.79 ± 0.00	0.31 ± 0.00
		LSTM	30	23.12 ± 0.43	16.60 ± 0.66	11.10 ± 0.63	87.19 ± 0.47	0.74 ± 0.01	0.86 ± 0.01	0.24 ± 0.01
			60	36.08 ± 1.47	25.38 ± 0.84	16.62 ± 0.25	68.60 ± 2.56	0.59 ± 0.02	0.75 ± 0.01	0.34 ± 0.01
	563	MLP	30	18.71 ± 0.05	13.46 ± 0.06	8.47 ± 0.04	82.97 ± 0.09	0.74 ± 0.00	0.91 ± 0.00	0.19 ± 0.00
			60	30.65 ± 0.01	21.69 ± 0.04	13.46 ± 0.04	54.36 ± 0.04	0.57 ± 0.01	0.81 ± 0.00	0.30 ± 0.00
		LSTM	30	21.59 ± 0.64	15.33 ± 0.45	9.69 ± 0.19	77.31 ± 1.34	0.72 ± 0.01	0.89 ± 0.00	0.22 ± 0.00
			60	33.02 ± 0.62	24.13 ± 0.61	15.07 ± 0.18	47.03 ± 2.01	0.51 ± 0.01	0.75 ± 0.02	0.33 ± 0.01
	570	MLP	30	17.44 ± 0.03	12.47 ± 0.03	6.38 ± 0.03	93.34 ± 0.03	0.86 ± 0.00	0.96 ± 0.00	0.12 ± 0.00
			60	29.00 ± 0.14	20.97 ± 0.13	10.73 ± 0.04	81.62 ± 0.18	0.79 ± 0.00	0.91 ± 0.00	0.20 ± 0.00
		LSTM	30	22.92 ± 1.49	16.16 ± 1.15	8.04 ± 0.65	88.47 ± 1.52	0.81 ± 0.02	0.94 ± 0.01	0.15 ± 0.01
			60	35.80 ± 1.50	26.75 ± 1.85	12.68 ± 0.43	71.95 ± 2.31	0.75 ± 0.00	0.88 ± 0.01	0.23 ± 0.01
	575	MLP	30	24.12 ± 0.06	16.05 ± 0.10	11.43 ± 0.09	84.48 ± 0.07	0.73 ± 0.00	0.86 ± 0.00	0.24 ± 0.00
			60	35.63 ± 0.17	25.66 ± 0.20	18.91 ± 0.17	66.19 ± 0.32	0.57 ± 0.01	0.71 ± 0.00	0.38 ± 0.00
		LSTM	30	27.20 ± 0.57	18.25 ± 0.45	13.14 ± 0.71	80.24 ± 0.82	0.69 ± 0.00	0.82 ± 0.02	0.28 ± 0.01
			60	38.09 ± 0.03	27.47 ± 0.52	20.48 ± 1.20	61.36 ± 0.07	0.54 ± 0.02	0.70 ± 0.00	0.41 ± 0.01
	588	MLP	30	18.07 ± 0.35	13.50 ± 0.15	8.29 ± 0.01	85.66 ± 0.56	0.76 ± 0.01	0.93 ± 0.00	0.18 ± 0.00
			60	30.36 ± 0.11	22.68 ± 0.13	14.16 ± 0.12	59.60 ± 0.28	0.58 ± 0.00	0.77 ± 0.00	0.31 ± 0.00
		LSTM	30	19.23 ± 0.11	14.16 ± 0.11	8.53 ± 0.12	83.77 ± 0.19	0.74 ± 0.00	0.92 ± 0.00	0.19 ± 0.00
			60	30.46 ± 0.60	22.48 ± 0.39	14.04 ± 0.23	59.33 ± 1.61	0.60 ± 0.01	0.79 ± 0.01	0.30 ± 0.01
	591	MLP	30	22.98 ± 0.11	16.61 ± 0.05	12.99 ± 0.03	80.32 ± 0.18	0.65 ± 0.01	0.80 ± 0.00	0.29 ± 0.00
			60	34.98 ± 0.05	26.93 ± 0.08	21.91 ± 0.13	54.41 ± 0.12	0.39 ± 0.00	0.65 ± 0.00	0.45 ± 0.00
		LSTM	30	26.33 ± 0.42	19.55 ± 0.24	15.65 ± 0.40	74.16 ± 0.83	0.60 ± 0.00	0.75 ± 0.01	0.34 ± 0.01
			60	36.51 ± 0.20	28.36 ± 0.26	23.32 ± 0.27	50.32 ± 0.54	0.37 ± 0.02	0.63 ± 0.00	0.47 ± 0.00
2020	540	MLP	30	22.88 ± 0.13	17.45 ± 0.10	12.71 ± 0.04	87.60 ± 0.14	0.68 ± 0.00	0.81 ± 0.00	0.27 ± 0.00
			60	39.84 ± 0.14	30.49 ± 0.12	22.96 ± 0.13	62.48 ± 0.27	0.52 ± 0.00	0.66 ± 0.00	0.44 ± 0.00
		LSTM	30	24.84 ± 0.42	18.48 ± 0.70	13.81 ± 1.24	85.37 ± 0.49	0.67 ± 0.02	0.80 ± 0.01	0.29 ± 0.02
			60	41.36 ± 0.58	30.69 ± 0.37	22.40 ± 0.20	59.56 ± 1.12	0.50 ± 0.02	0.66 ± 0.00	0.44 ± 0.00
	544	MLP	30	17.37 ± 0.03	12.14 ± 0.03	8.21 ± 0.03	88.26 ± 0.04	0.78 ± 0.00	0.92 ± 0.00	0.18 ± 0.00
			60	28.49 ± 0.03	20.74 ± 0.04	14.16 ± 0.05	68.32 ± 0.07	0.63 ± 0.00	0.78 ± 0.00	0.30 ± 0.00
		LSTM	30	21.23 ± 0.53	15.00 ± 0.49	9.93 ± 0.35	82.45 ± 0.87	0.76 ± 0.01	0.89 ± 0.00	0.21 ± 0.01
			60	30.45 ± 0.12	22.09 ± 0.45	14.81 ± 0.52	63.83 ± 0.29	0.59 ± 0.02	0.78 ± 0.01	0.31 ± 0.01
	552	MLP	30	14.06 ± 0.03	8.25 ± 0.11	6.48 ± 0.09	86.18 ± 0.05	0.75 ± 0.00	0.92 ± 0.00	0.14 ± 0.00
			60	23.83 ± 0.03	14.57 ± 0.10	11.75 ± 0.12	60.36 ± 0.09	0.64 ± 0.00	0.84 ± 0.00	0.22 ± 0.00
		LSTM	30	16.72 ± 0.44	10.31 ± 0.24	8.04 ± 0.22	80.45 ± 1.01	0.71 ± 0.02	0.90 ± 0.01	0.16 ± 0.01
			60	25.47 ± 0.30	16.27 ± 0.24	13.02 ± 0.27	54.73 ± 1.05	0.61 ± 0.01	0.83 ± 0.01	0.24 ± 0.01
	567	MLP	30	22.72 ± 0.04	16.47 ± 0.04	12.48 ± 0.03	84.80 ± 0.05	0.64 ± 0.00	0.80 ± 0.00	0.28 ± 0.00
			60	38.38 ± 0.02	29.51 ± 0.04	23.24 ± 0.06	56.68 ± 0.04	0.46 ± 0.00	0.64 ± 0.00	0.47 ± 0.00
		LSTM	30	24.64 ± 0.97	17.85 ± 0.81	13.48 ± 0.66	82.10 ± 1.41	0.60 ± 0.01	0.78 ± 0.01	0.31 ± 0.01
			60	40.13 ± 1.22	30.57 ± 1.14	25.05 ± 1.96	52.61 ± 2.86	0.45 ± 0.01	0.62 ± 0.02	0.50 ± 0.03
	584	MLP	30	22.78 ± 0.04	16.92 ± 0.04	11.34 ± 0.03	85.49 ± 0.05	0.77 ± 0.00	0.87 ± 0.00	0.23 ± 0.00
			60	35.99 ± 0.05	27.29 ± 0.02	18.40 ± 0.03	63.67 ± 0.11	0.60 ± 0.00	0.72 ± 0.00	0.37 ± 0.00
		LSTM	30	25.31 ± 1.32	18.27 ± 0.95	11.49 ± 0.52	82.05 ± 1.89	0.75 ± 0.01	0.86 ± 0.01	0.23 ± 0.01
			60	41.45 ± 1.58	31.50 ± 1.91	21.43 ± 2.17	51.75 ± 3.64	0.55 ± 0.03	0.67 ± 0.04	0.42 ± 0.04
	596	MLP	30	17.87 ± 0.08	12.89 ± 0.06	9.67 ± 0.03	86.99 ± 0.12	0.74 ± 0.00	0.89 ± 0.00	0.20 ± 0.00
			60	35.99 ± 0.05	27.29 ± 0.02	18.40 ± 0.03	63.67 ± 0.11	0.60 ± 0.00	0.72 ± 0.00	0.37 ± 0.00
		LSTM	30	19.96 ± 0.28	14.31 ± 0.03	10.83 ± 0.18	83.78 ± 0.45	0.70 ± 0.01	0.87 ± 0.00	0.23 ± 0.00
			60	30.28 ± 0.72	22.17 ± 0.71	16.97 ± 0.45	62.72 ± 1.77	0.56 ± 0.02	0.79 ± 0.00	0.32 ± 0.01

Note. PID: patient identification; PH: prediction horizon; LL: lag length; RMSE: root mean square error; SD: standard deviation; MAE: mean absolute error; MAPE: mean absolute percentage error; r^2^: coefficient of determination; MCC: Matthew’s correlation coefficient; SE: surveillance error; ASE: average surveillance error.

**Table 3:** The evaluation results for the stacking models created using Ohio datasets.

Dataset	PID	Learner	PH	Evaluation Metric						
				RMSE ± SD (mg/dL)	MAE ± SD (mg/dL)	MAPE ± SD (%)	r^2^ ± SD (%)	MCC ± SD (%)	SE < 0.5 ± SD (%)	ASE ± SD
2018	559	MLP	30	19.00 ± 0.11	13.19 ± 0.08	8.79 ± 0.05	91.35 ± 0.10	0.78 ± 0.00	0.90 ± 0.00	0.19 ± 0.00
			60	31.25 ± 0.41	22.67 ± 0.22	15.22 ± 0.24	76.46 ± 0.61	0.64 ± 0.00	0.79 ± 0.00	0.31 ± 0.00
		LSTM	30	22.90 ± 0.49	15.77 ± 0.17	9.97 ± 0.09	87.43 ± 0.54	0.76 ± 0.01	0.89 ± 0.00	0.21 ± 0.00
			60	34.95 ± 0.17	24.99 ± 0.11	16.61 ± 0.05	70.56 ± 0.29	0.61 ± 0.01	0.76 ± 0.00	0.33 ± 0.00
	563	MLP	30	18.54 ± 0.05	13.03 ± 0.03	8.10 ± 0.00	83.28 ± 0.08	0.74 ± 0.01	0.92 ± 0.00	0.18 ± 0.00
			60	29.87 ± 0.18	21.22 ± 0.14	13.36 ± 0.04	56.67 ± 0.51	0.58 ± 0.01	0.81 ± 0.00	0.30 ± 0.00
		LSTM	30	21.25 ± 0.05	14.97 ± 0.06	9.38 ± 0.02	78.05 ± 0.11	0.73 ± 0.00	0.89 ± 0.00	0.21 ± 0.00
			60	33.20 ± 0.16	23.55 ± 0.07	14.44 ± 0.02	46.46 ± 0.53	0.52 ± 0.00	0.78 ± 0.00	0.32 ± 0.00
	570	MLP	30	17.49 ± 0.11	12.43 ± 0.10	6.36 ± 0.03	93.30 ± 0.09	0.86 ± 0.01	0.96 ± 0.00	0.12 ± 0.00
			60	28.65 ± 0.08	20.90 ± 0.07	10.91 ± 0.04	82.06 ± 0.10	0.78 ± 0.00	0.91 ± 0.00	0.20 ± 0.00
		LSTM	30	21.58 ± 1.50	15.59 ± 1.55	7.70 ± 0.49	89.77 ± 1.44	0.84 ± 0.01	0.94 ± 0.00	0.14 ± 0.01
			60	32.48 ± 0.69	23.55 ± 0.62	11.82 ± 0.06	76.93 ± 0.98	0.76 ± 0.00	0.89 ± 0.00	0.22 ± 0.00
	575	MLP	30	24.21 ± 0.04	15.70 ± 0.09	11.25 ± 0.19	84.36 ± 0.05	0.74 ± 0.00	0.86 ± 0.00	0.24 ± 0.00
			60	36.42 ± 0.41	26.35 ± 0.77	19.85 ± 1.57	64.68 ± 0.79	0.57 ± 0.02	0.71 ± 0.00	0.40 ± 0.02
		LSTM	30	27.73 ± 0.12	18.09 ± 0.09	12.67 ± 0.09	79.48 ± 0.18	0.66 ± 0.00	0.82 ± 0.00	0.27 ± 0.00
			60	38.34 ± 0.09	27.48 ± 0.06	19.59 ± 0.12	60.86 ± 0.18	0.54 ± 0.00	0.68 ± 0.00	0.41 ± 0.00
	588	MLP	30	18.24 ± 0.19	13.51 ± 0.12	8.17 ± 0.02	85.39 ± 0.30	0.75 ± 0.01	0.93 ± 0.00	0.18 ± 0.00
			60	29.65 ± 0.21	21.84 ± 0.18	13.14 ± 0.08	61.46 ± 0.55	0.57 ± 0.01	0.80 ± 0.00	0.29 ± 0.00
		LSTM	30	18.91 ± 0.08	14.03 ± 0.14	8.43 ± 0.25	84.30 ± 0.13	0.75 ± 0.00	0.92 ± 0.00	0.18 ± 0.01
			60	30.67 ± 0.20	22.29 ± 0.25	13.54 ± 0.49	58.76 ± 0.54	0.60 ± 0.01	0.81 ± 0.01	0.29 ± 0.01
	591	MLP	30	22.88 ± 0.07	16.60 ± 0.04	13.03 ± 0.06	80.49 ± 0.12	0.65 ± 0.00	0.80 ± 0.00	0.29 ± 0.00
			60	34.43 ± 0.06	26.80 ± 0.05	22.09 ± 0.09	55.84 ± 0.14	0.41 ± 0.00	0.65 ± 0.00	0.45 ± 0.00
		LSTM	30	25.51 ± 0.01	18.80 ± 0.05	14.79 ± 0.08	75.73 ± 0.03	0.59 ± 0.00	0.76 ± 0.00	0.33 ± 0.00
			60	36.68 ± 0.16	28.44 ± 0.05	23.78 ± 0.03	49.87 ± 0.44	0.42 ± 0.00	0.64 ± 0.00	0.47 ± 0.00
2020	540	MLP	30	22.34 ± 0.02	17.13 ± 0.03	12.58 ± 0.03	88.18 ± 0.02	0.68 ± 0.00	0.82 ± 0.00	0.27 ± 0.00
			60	39.40 ± 0.09	30.32 ± 0.13	22.95 ± 0.10	63.29 ± 0.17	0.52 ± 0.00	0.66 ± 0.00	0.44 ± 0.00
		LSTM	30	24.13 ± 0.14	18.24 ± 0.06	13.57 ± 0.03	86.20 ± 0.17	0.66 ± 0.00	0.80 ± 0.00	0.29 ± 0.00
			60	40.86 ± 0.05	30.62 ± 0.11	23.06 ± 0.18	60.53 ± 0.09	0.51 ± 0.00	0.66 ± 0.00	0.44 ± 0.00
	544	MLP	30	16.96 ± 0.02	12.01 ± 0.05	8.14 ± 0.08	88.81 ± 0.03	0.79 ± 0.00	0.92 ± 0.00	0.18 ± 0.00
			60	28.36 ± 0.17	20.72 ± 0.04	14.21 ± 0.08	68.62 ± 0.37	0.64 ± 0.00	0.78 ± 0.00	0.30 ± 0.00
		LSTM	30	20.85 ± 0.25	14.84 ± 0.20	10.01 ± 0.14	83.08 ± 0.40	0.73 ± 0.00	0.88 ± 0.00	0.22 ± 0.00
			60	31.30 ± 0.23	22.55 ± 0.10	15.44 ± 0.07	61.77 ± 0.57	0.59 ± 0.00	0.76 ± 0.00	0.33 ± 0.00
	552	MLP	30	14.19 ± 0.03	9.00 ± 0.06	7.10 ± 0.03	85.92 ± 0.05	0.72 ± 0.00	0.91 ± 0.00	0.15 ± 0.00
			60	23.78 ± 0.04	15.52 ± 0.20	12.62 ± 0.18	60.53 ± 0.14	0.61 ± 0.01	0.84 ± 0.00	0.23 ± 0.00
		LSTM	30	17.65 ± 0.22	11.92 ± 0.20	9.79 ± 0.21	78.23 ± 0.53	0.69 ± 0.00	0.88 ± 0.01	0.19 ± 0.01
			60	26.93 ± 0.23	17.97 ± 0.17	15.04 ± 0.14	49.39 ± 0.85	0.58 ± 0.01	0.78 ± 0.00	0.28 ± 0.00
	567	MLP	30	22.67 ± 0.22	16.17 ± 0.22	12.39 ± 0.21	84.86 ± 0.29	0.64 ± 0.01	0.81 ± 0.00	0.28 ± 0.00
			60	37.82 ± 0.24	28.14 ± 0.18	22.42 ± 0.23	57.94 ± 0.52	0.48 ± 0.00	0.66 ± 0.00	0.46 ± 0.00
		LSTM	30	23.74 ± 0.09	16.86 ± 0.14	12.96 ± 0.14	83.41 ± 0.13	0.62 ± 0.00	0.79 ± 0.00	0.30 ± 0.00
			60	38.75 ± 0.41	29.24 ± 0.31	23.40 ± 0.46	55.84 ± 0.92	0.47 ± 0.01	0.64 ± 0.01	0.48 ± 0.01
	584	MLP	30	21.89 ± 0.09	15.96 ± 0.14	10.64 ± 0.13	86.60 ± 0.11	0.77 ± 0.00	0.89 ± 0.00	0.22 ± 0.00
			60	35.42 ± 0.42	26.73 ± 0.52	17.97 ± 0.53	64.79 ± 0.83	0.60 ± 0.01	0.73 ± 0.01	0.36 ± 0.01
		LSTM	30	24.79 ± 0.06	18.21 ± 0.08	12.51 ± 0.13	82.82 ± 0.08	0.76 ± 0.00	0.86 ± 0.00	0.25 ± 0.00
			60	38.65 ± 0.29	29.33 ± 0.12	20.14 ± 0.01	58.09 ± 0.63	0.60 ± 0.00	0.70 ± 0.00	0.39 ± 0.00
	596	MLP	30	17.76 ± 0.09	12.85 ± 0.09	9.71 ± 0.11	87.16 ± 0.13	0.75 ± 0.00	0.90 ± 0.00	0.20 ± 0.00
			60	28.80 ± 0.19	21.37 ± 0.13	16.53 ± 0.11	66.29 ± 0.44	0.59 ± 0.01	0.80 ± 0.00	0.31 ± 0.00
		LSTM	30	19.06 ± 0.16	13.55 ± 0.08	10.27 ± 0.06	85.21 ± 0.24	0.72 ± 0.00	0.88 ± 0.00	0.22 ± 0.00
			60	30.01 ± 0.10	22.25 ± 0.10	17.31 ± 0.16	63.39 ± 0.25	0.56 ± 0.00	0.80 ± 0.00	0.32 ± 0.00

Note. PID: patient identification; PH: prediction horizon; LL: lag length; RMSE: root mean square error; SD: standard deviation; MAE: mean absolute error; MAPE: mean absolute percentage error; r^2^: coefficient of determination; MCC: Matthew’s correlation coefficient; SE: surveillance error; ASE: average surveillance error.

**Table 4:** The evaluation results for the nested stacking models created using Ohio datasets.

Dataset	PID	Learner	PH	Evaluation Metric						
				RMSE ± SD (mg/dL)	MAE ± SD (mg/dL)	MAPE ± SD (%)	r^2^ ± SD (%)	MCC ± SD (%)	SE < 0.5 ± SD (%)	ASE ± SD
2018	559	MLP	30	19.67 ± 0.05	13.54 ± 0.05	8.89 ± 0.03	90.72 ± 0.05	0.79 ± 0.00	0.90 ± 0.00	0.19 ± 0.00
			60	33.44 ± 0.28	23.54 ± 0.16	15.27 ± 0.04	73.05 ± 0.46	0.63 ± 0.00	0.78 ± 0.00	0.31 ± 0.00
		LSTM	30	19.69 ± 0.19	13.51 ± 0.18	8.83 ± 0.17	90.71 ± 0.18	0.79 ± 0.00	0.90 ± 0.00	0.19 ± 0.00
			60	33.93 ± 0.48	23.82 ± 0.28	15.31 ± 0.05	72.25 ± 0.79	0.63 ± 0.01	0.78 ± 0.00	0.31 ± 0.00
	563	MLP	30	18.85 ± 0.10	13.15 ± 0.08	8.27 ± 0.02	82.72 ± 0.19	0.76 ± 0.01	0.91 ± 0.00	0.18 ± 0.00
			60	31.82 ± 0.54	22.38 ± 0.38	13.84 ± 0.11	50.81 ± 1.66	0.55 ± 0.01	0.80 ± 0.01	0.30 ± 0.00
		LSTM	30	19.00 ± 0.07	13.24 ± 0.06	8.31 ± 0.03	82.44 ± 0.13	0.76 ± 0.01	0.91 ± 0.00	0.19 ± 0.00
			60	31.65 ± 0.51	22.37 ± 0.61	13.79 ± 0.10	51.35 ± 1.59	0.55 ± 0.03	0.80 ± 0.01	0.31 ± 0.01
	570	MLP	30	18.34 ± 0.11	12.85 ± 0.08	6.58 ± 0.05	92.64 ± 0.09	0.86 ± 0.00	0.96 ± 0.00	0.12 ± 0.00
			60	31.09 ± 0.28	22.21 ± 0.14	11.54 ± 0.03	78.88 ± 0.38	0.77 ± 0.00	0.89 ± 0.00	0.21 ± 0.00
		LSTM	30	18.57 ± 0.22	13.11 ± 0.12	6.65 ± 0.08	92.45 ± 0.18	0.86 ± 0.00	0.96 ± 0.00	0.12 ± 0.00
			60	31.61 ± 0.60	22.60 ± 0.54	11.53 ± 0.02	78.16 ± 0.84	0.77 ± 0.00	0.90 ± 0.00	0.21 ± 0.00
	575	MLP	30	26.18 ± 0.09	16.60 ± 0.19	12.40 ± 0.27	81.71 ± 0.12	0.73 ± 0.00	0.84 ± 0.00	0.26 ± 0.01
			60	36.98 ± 0.33	26.43 ± 0.50	19.46 ± 1.39	63.57 ± 0.65	0.54 ± 0.01	0.70 ± 0.01	0.40 ± 0.02
		LSTM	30	26.01 ± 0.91	16.47 ± 0.32	12.02 ± 0.66	81.93 ± 1.25	0.73 ± 0.00	0.84 ± 0.01	0.25 ± 0.01
			60	37.05 ± 0.62	26.29 ± 0.28	18.96 ± 0.13	63.44 ± 1.22	0.54 ± 0.00	0.70 ± 0.00	0.39 ± 0.00
	588	MLP	30	18.50 ± 0.11	13.63 ± 0.08	8.11 ± 0.05	84.98 ± 0.17	0.74 ± 0.00	0.93 ± 0.00	0.18 ± 0.00
			60	29.43 ± 0.07	21.42 ± 0.17	13.01 ± 0.42	62.05 ± 0.17	0.62 ± 0.00	0.82 ± 0.01	0.28 ± 0.01
		LSTM	30	18.26 ± 0.14	13.56 ± 0.27	8.23 ± 0.32	85.37 ± 0.22	0.76 ± 0.01	0.93 ± 0.00	0.18 ± 0.01
			60	29.54 ± 0.28	21.33 ± 0.21	12.84 ± 0.09	61.77 ± 0.74	0.62 ± 0.01	0.82 ± 0.00	0.27 ± 0.00
	591	MLP	30	23.07 ± 0.09	16.48 ± 0.04	12.89 ± 0.06	80.16 ± 0.15	0.64 ± 0.01	0.80 ± 0.00	0.29 ± 0.00
			60	35.68 ± 0.11	27.65 ± 0.08	23.12 ± 0.07	52.56 ± 0.29	0.42 ± 0.00	0.65 ± 0.00	0.46 ± 0.00
		LSTM	30	23.08 ± 0.10	16.52 ± 0.07	12.98 ± 0.08	80.14 ± 0.17	0.63 ± 0.00	0.80 ± 0.00	0.29 ± 0.00
			60	35.68 ± 0.21	27.69 ± 0.12	23.16 ± 0.08	52.57 ± 0.55	0.42 ± 0.00	0.65 ± 0.01	0.46 ± 0.00
2020	540	MLP	30	22.36 ± 0.03	16.96 ± 0.05	12.59 ± 0.03	88.15 ± 0.03	0.67 ± 0.00	0.82 ± 0.00	0.27 ± 0.00
			60	38.81 ± 0.26	29.34 ± 0.14	22.04 ± 0.10	64.38 ± 0.47	0.53 ± 0.01	0.68 ± 0.00	0.43 ± 0.00
		LSTM	30	22.39 ± 0.11	16.99 ± 0.09	12.61 ± 0.08	88.12 ± 0.12	0.67 ± 0.01	0.81 ± 0.00	0.27 ± 0.00
			60	38.74 ± 0.18	29.32 ± 0.18	22.05 ± 0.15	64.52 ± 0.33	0.53 ± 0.01	0.68 ± 0.00	0.43 ± 0.00
	544	MLP	30	16.86 ± 0.11	11.89 ± 0.06	8.02 ± 0.06	88.94 ± 0.14	0.78 ± 0.00	0.92 ± 0.00	0.17 ± 0.00
			60	28.92 ± 0.14	20.88 ± 0.05	14.33 ± 0.02	67.36 ± 0.31	0.63 ± 0.00	0.77 ± 0.00	0.30 ± 0.00
		LSTM	30	16.96 ± 0.15	11.95 ± 0.11	8.07 ± 0.09	88.80 ± 0.19	0.78 ± 0.01	0.92 ± 0.00	0.18 ± 0.00
			60	28.84 ± 0.19	20.81 ± 0.10	14.34 ± 0.13	67.54 ± 0.42	0.63 ± 0.00	0.77 ± 0.00	0.30 ± 0.00
	552	MLP	30	13.87 ± 0.16	8.88 ± 0.32	7.07 ± 0.24	86.56 ± 0.32	0.72 ± 0.01	0.92 ± 0.00	0.15 ± 0.01
			60	24.61 ± 0.11	16.04 ± 0.36	13.43 ± 0.30	57.73 ± 0.38	0.60 ± 0.00	0.82 ± 0.00	0.25 ± 0.00
		LSTM	30	13.86 ± 0.02	9.00 ± 0.06	7.13 ± 0.06	86.58 ± 0.03	0.72 ± 0.00	0.92 ± 0.00	0.15 ± 0.00
			60	23.97 ± 0.44	15.47 ± 0.32	12.76 ± 0.38	59.91 ± 1.47	0.61 ± 0.00	0.83 ± 0.01	0.24 ± 0.01
	567	MLP	30	21.81 ± 0.28	15.58 ± 0.14	11.71 ± 0.30	86.00 ± 0.35	0.65 ± 0.01	0.82 ± 0.01	0.27 ± 0.01
			60	37.50 ± 0.18	27.95 ± 0.13	21.97 ± 0.18	58.65 ± 0.39	0.49 ± 0.00	0.66 ± 0.00	0.46 ± 0.00
		LSTM	30	22.02 ± 0.07	15.70 ± 0.05	11.96 ± 0.07	85.72 ± 0.08	0.64 ± 0.00	0.82 ± 0.00	0.27 ± 0.00
			60	37.77 ± 0.25	28.19 ± 0.22	22.38 ± 0.36	58.05 ± 0.55	0.48 ± 0.00	0.66 ± 0.00	0.46 ± 0.00
	584	MLP	30	22.35 ± 0.58	16.74 ± 0.67	11.54 ± 0.54	86.03 ± 0.73	0.77 ± 0.01	0.88 ± 0.01	0.24 ± 0.01
			60	35.77 ± 0.49	27.25 ± 0.49	18.79 ± 0.44	64.11 ± 0.99	0.61 ± 0.01	0.73 ± 0.01	0.37 ± 0.01
		LSTM	30	22.19 ± 0.11	16.54 ± 0.17	11.38 ± 0.17	86.24 ± 0.13	0.77 ± 0.00	0.88 ± 0.00	0.23 ± 0.00
			60	36.02 ± 0.06	27.37 ± 0.12	18.91 ± 0.14	63.60 ± 0.12	0.61 ± 0.00	0.72 ± 0.00	0.37 ± 0.00
	596	MLP	30	17.78 ± 0.24	12.67 ± 0.13	9.52 ± 0.10	87.13 ± 0.35	0.74 ± 0.00	0.89 ± 0.00	0.20 ± 0.00
			60	28.54 ± 0.24	20.79 ± 0.09	15.74 ± 0.27	66.89 ± 0.55	0.58 ± 0.02	0.81 ± 0.00	0.30 ± 0.00
		LSTM	30	17.57 ± 0.25	12.49 ± 0.14	9.35 ± 0.09	87.43 ± 0.36	0.75 ± 0.01	0.89 ± 0.00	0.20 ± 0.00
			60	28.68 ± 0.37	20.97 ± 0.07	15.96 ± 0.31	66.55 ± 0.87	0.58 ± 0.02	0.81 ± 0.00	0.31 ± 0.00

Note. PID: patient identification; PH: prediction horizon; LL: lag length; RMSE: root mean square error; SD: standard deviation; MAE: mean absolute error; MAPE: mean absolute percentage error; r^2^: coefficient of determination; MCC: Matthew’s correlation coefficient; SE: surveillance error; ASE: average surveillance error.

## Data Availability

The Ohio datasets used in this research are publicly accessible upon request by following the instructions provided in this link.

## Appendix A

In this section, the complete outcomes of evaluation analysis on the non-stacking models are provided in four tables, as below.

**Table A1:** The evaluation results for non-stacking models created by multilayer perceptron learners using Ohio 2018 dataset.

PID	PH	LL	Evaluation metric						
			RMSE ± SD (mg/dL)	MAE ± SD (mg/dL)	MAPE ± SD (%)	r^2^ ± SD (%)	MCC ± SD (%)	SE < 0.5 ± SD (%)	ASE ± SD
559	30	30	19.96 ± 0.09	13.78 ± 0.11	8.83 ± 0.11	90.45 ± 0.08	**0.77 ± 0.00**	**0.90 ± 0.00**	**0.19 ± 0.00**
		60	**19.65 ± 0.06**	**13.56 ± 0.03**	**8.78 ± 0.03**	**90.75 ± 0.05**	**0.77 ± 0.00**	**0.90 ± 0.00**	**0.19 ± 0.00**
		90	19.85 ± 0.01	13.73 ± 0.02	8.81 ± 0.04	90.56 ± 0.01	**0.77 ± 0.00**	**0.90 ± 0.00**	**0.19 ± 0.00**
		120	19.88 ± 0.07	13.83 ± 0.05	8.81 ± 0.04	90.53 ± 0.07	**0.77 ± 0.00**	**0.90 ± 0.00**	**0.19 ± 0.00**
	60	30	33.73 ± 0.04	24.46 ± 0.04	16.49 ± 0.05	72.59 ± 0.06	0.58 ± 0.00	0.77 ± 0.00	0.33 ± 0.00
		60	32.04 ± 0.05	23.12 ± 0.09	15.43 ± 0.11	75.26 ± 0.08	0.62 ± 0.01	**0.79 ± 0.00**	**0.31 ± 0.00**
		90	31.67 ± 0.05	22.84 ± 0.06	15.23 ± 0.04	75.82 ± 0.08	**0.64 ± 0.00**	**0.79 ± 0.00**	**0.31 ± 0.00**
		120	**31.36 ± 0.06**	**22.78 ± 0.06**	**15.18 ± 0.07**	**76.30 ± 0.08**	0.63 ± 0.00	**0.79 ± 0.00**	**0.31 ± 0.00**
563	30	30	**18.71 ± 0.05**	13.46 ± 0.06	8.47 ± 0.04	**82.97 ± 0.09**	0.74 ± 0.00	**0.91 ± 0.00**	**0.19 ± 0.00**
		60	18.89 ± 0.03	**13.33 ± 0.03**	**8.30 ± 0.02**	82.65 ± 0.05	0.74 ± 0.00	**0.91 ± 0.00**	**0.19 ± 0.00**
		90	19.09 ± 0.03	13.42 ± 0.03	8.34 ± 0.02	82.27 ± 0.06	**0.74 ± 0.01**	**0.91 ± 0.00**	**0.19 ± 0.00**
		120	19.29 ± 0.01	13.61 ± 0.00	8.45 ± 0.00	81.91 ± 0.02	0.73 ± 0.01	**0.91 ± 0.00**	**0.19 ± 0.00**
	60	30	30.44 ± 0.08	22.46 ± 0.08	14.40 ± 0.06	55.00 ± 0.23	0.49 ± 0.00	0.78 ± 0.00	0.33 ± 0.00
		60	**30.43 ± 0.05**	21.75 ± 0.02	13.57 ± 0.02	**55.02 ± 0.14**	0.56 ± 0.01	0.80 ± 0.00	**0.30 ± 0.00**
		90	30.65 ± 0.01	**21.69 ± 0.04**	**13.46 ± 0.04**	54.36 ± 0.04	**0.57 ± 0.01**	**0.81 ± 0.00**	**0.30 ± 0.00**
		120	30.68 ± 0.15	21.72 ± 0.09	13.47 ± 0.05	54.28 ± 0.44	0.57 ± 0.00	**0.81 ± 0.00**	**0.30 ± 0.00**
570	30	30	18.24 ± 0.19	13.27 ± 0.15	6.74 ± 0.08	92.71 ± 0.15	0.84 ± 0.00	0.95 ± 0.00	0.13 ± 0.00
		60	**17.44 ± 0.03**	**12.47 ± 0.03**	**6.38 ± 0.03**	**93.34 ± 0.03**	**0.86 ± 0.00**	**0.96 ± 0.00**	**0.12 ± 0.00**
		90	17.58 ± 0.03	12.54 ± 0.03	6.45 ± 0.01	**93.24 ± 0.03**	**0.86 ± 0.00**	**0.96 ± 0.00**	**0.12 ± 0.00**
		120	17.71 ± 0.13	12.53 ± 0.11	6.41 ± 0.06	93.13 ± 0.10	**0.86 ± 0.00**	**0.96 ± 0.00**	**0.12 ± 0.00**
	60	30	30.36 ± 0.08	23.08 ± 0.07	11.89 ± 0.03	79.85 ± 0.10	0.74 ± 0.00	0.89 ± 0.00	0.22 ± 0.00
		60	**28.89 ± 0.03**	21.33 ± 0.04	10.92 ± 0.01	**81.76 ± 0.04**	0.78 ± 0.00	**0.91 ± 0.00**	**0.20 ± 0.00**
		90	28.95 ± 0.10	**21.07 ± 0.09**	10.82 ± 0.02	81.68 ± 0.13	**0.79 ± 0.00**	**0.91 ± 0.00**	**0.20 ± 0.00**
		120	29.00 ± 0.14	20.97 ± 0.13	**10.73 ± 0.04**	81.62 ± 0.18	**0.79 ± 0.00**	**0.91 ± 0.00**	**0.20 ± 0.00**
575	30	30	**24.12 ± 0.06**	16.05 ± 0.10	11.43 ± 0.09	**84.48 ± 0.07**	0.73 ± 0.00	**0.86 ± 0.00**	0.24 ± 0.00
		60	24.49 ± 0.04	**15.93 ± 0.02**	**11.39 ± 0.02**	84.00 ± 0.06	0.73 ± 0.00	0.85 ± 0.00	**0.25 ± 0.00**
		90	24.38 ± 0.09	15.97 ± 0.13	11.56 ± 0.11	84.13 ± 0.12	0.74 ± 0.00	0.85 ± 0.00	**0.25 ± 0.00**
		120	24.35 ± 0.09	16.07 ± 0.12	11.72 ± 0.16	84.17 ± 0.12	**0.75 ± 0.00**	0.85 ± 0.01	**0.25 ± 0.00**
	60	30	36.22 ± 0.10	26.77 ± 0.12	19.49 ± 0.10	65.08 ± 0.19	0.51 ± 0.00	0.69 ± 0.00	0.40 ± 0.00
		60	36.27 ± 0.20	26.24 ± 0.25	18.96 ± 0.17	64.96 ± 0.39	0.54 ± 0.01	0.70 ± 0.00	0.39 ± 0.00
		90	35.90 ± 0.23	25.73 ± 0.11	**18.79 ± 0.09**	65.68 ± 0.44	0.55 ± 0.00	0.70 ± 0.00	0.39 ± 0.00
		120	**35.63 ± 0.17**	**25.66 ± 0.20**	18.91 ± 0.17	**66.19 ± 0.32**	**0.57 ± 0.01**	**0.71 ± 0.00**	0.38 ± 0.00
588	30	30	18.80 ± 0.09	13.99 ± 0.09	8.63 ± 0.07	84.49 ± 0.15	**0.75 ± 0.00**	0.92 ± 0.00	0.19 ± 0.00
		60	18.27 ± 0.42	13.61 ± 0.20	8.36 ± 0.06	85.35 ± 0.68	0.75 ± 0.02	**0.93 ± 0.00**	**0.18 ± 0.00**
		90	**18.07 ± 0.35**	**13.50 ± 0.15**	8.29 ± 0.01	85.66 ± 0.56	0.76 ± 0.01	**0.93 ± 0.00**	**0.18 ± 0.00**
		120	18.44 ± 0.67	13.64 ± 0.37	**8.26 ± 0.13**	**85.06 ± 1.09**	0.75 ± 0.02	**0.93 ± 0.01**	**0.18 ± 0.00**
	60	30	**30.36 ± 0.11**	22.68 ± 0.13	14.16 ± 0.12	**59.60 ± 0.28**	**0.58 ± 0.00**	0.77 ± 0.00	0.31 ± 0.00
		60	30.72 ± 0.26	22.76 ± 0.25	13.62 ± 0.16	58.65 ± 0.69	0.56 ± 0.01	0.79 ± 0.00	0.30 ± 0.00
		90	30.58 ± 0.05	**22.47 ± 0.10**	13.41 ± 0.08	59.01 ± 0.13	0.56 ± 0.00	**0.80 ± 0.00**	**0.29 ± 0.00**
		120	30.48 ± 0.25	22.39 ± 0.26	**13.33 ± 0.19**	59.29 ± 0.67	0.57 ± 0.01	**0.80 ± 0.00**	**0.29 ± 0.00**
591	30	30	**22.89 ± 0.02**	16.68 ± 0.02	**12.98 ± 0.02**	**80.47 ± 0.04**	0.62 ± 0.00	0.79 ± 0.00	**0.29 ± 0.00**
		60	22.98 ± 0.11	**16.61 ± 0.05**	12.99 ± 0.03	80.32 ± 0.18	**0.65 ± 0.01**	**0.80 ± 0.00**	**0.29 ± 0.00**
		90	23.01 ± 0.06	16.71 ± 0.01	13.12 ± 0.02	80.26 ± 0.09	0.64 ± 0.01	**0.80 ± 0.00**	**0.29 ± 0.00**
		120	22.97 ± 0.07	16.78 ± 0.05	13.21 ± 0.11	80.32 ± 0.12	0.64 ± 0.01	**0.80 ± 0.00**	**0.29 ± 0.00**
	60	30	35.00 ± 0.05	27.27 ± 0.06	22.01 ± 0.07	54.35 ± 0.14	0.36 ± 0.00	0.64 ± 0.00	**0.45 ± 0.00**
		60	35.93 ± 0.07	27.77 ± 0.02	22.37 ± 0.07	51.89 ± 0.19	0.35 ± 0.00	0.63 ± 0.00	0.46 ± 0.00
		90	34.98 ± 0.05	**26.93 ± 0.08**	**21.91 ± 0.13**	54.41 ± 0.12	**0.39 ± 0.00**	**0.65 ± 0.00**	**0.45 ± 0.00**
		120	**34.91 ± 0.07**	27.12 ± 0.16	22.19 ± 0.25	**54.60 ± 0.19**	**0.39 ± 0.00**	**0.65 ± 0.00**	**0.45 ± 0.00**

Note. Values in bold indicate the best evaluation outcome for each metric in each learning scenario, and grey highlights denote the best model in each scenario based on the best-achieved evaluation metrics. Note. PID: patient identification; PH: prediction horizon; LL: lag length; RMSE: root mean square error; SD: standard deviation; MAE: mean absolute error; MAPE: mean absolute percentage error; r^2^: coefficient of determination; MCC: Matthew’s correlation coefficient; SE: surveillance error; ASE: average surveillance error.

**Table A2:** The evaluation results for non-stacking models created by multilayer perceptron learners using Ohio 2020 dataset.

PID	PH	LL	Evaluation Metric						
			RMSE ± SD (mg/dL)	MAE ± SD (mg/dL)	MAPE ± SD (%)	r^2^ ± SD (%)	MCC ± SD (%)	SE < 0.5 ± SD (%)	ASE ± SD
540	30	30	23.48 ± 0.04	17.73 ± 0.03	12.88 ± 0.00	86.93 ± 0.04	0.67 ± 0.00	**0.81 ± 0.00**	**0.28 ± 0.00**
		60	**22.88 ± 0.13**	**17.45 ± 0.10**	**12.71 ± 0.04**	87.60 ± 0.14	**0.68 ± 0.00**	**0.81 ± 0.00**	0.27 ± 0.00
		90	23.41 ± 0.08	17.79 ± 0.04	12.84 ± 0.04	87.02 ± 0.09	**0.68 ± 0.00**	**0.81 ± 0.00**	**0.28 ± 0.00**
		120	23.61 ± 0.13	17.92 ± 0.07	12.86 ± 0.02	**86.79 ± 0.15**	0.67 ± 0.00	**0.81 ± 0.00**	**0.28 ± 0.00**
	60	30	40.74 ± 0.16	31.20 ± 0.15	23.55 ± 0.12	60.76 ± 0.32	0.49 ± 0.00	0.65 ± 0.00	0.45 ± 0.00
		60	**39.84 ± 0.14**	**30.49 ± 0.12**	**22.96 ± 0.13**	**62.48 ± 0.27**	**0.52 ± 0.00**	**0.66 ± 0.00**	**0.44 ± 0.00**
		90	40.15 ± 0.16	30.68 ± 0.15	23.09 ± 0.14	61.90 ± 0.30	0.52 ± 0.01	**0.66 ± 0.00**	**0.44 ± 0.00**
		120	40.38 ± 0.16	30.88 ± 0.14	23.16 ± 0.07	61.45 ± 0.31	**0.52 ± 0.00**	**0.66 ± 0.00**	**0.44 ± 0.00**
544	30	30	17.76 ± 0.06	12.45 ± 0.07	8.47 ± 0.07	87.73 ± 0.09	**0.78 ± 0.00**	0.91 ± 0.00	**0.18 ± 0.00**
		60	**17.37 ± 0.03**	**12.14 ± 0.03**	**8.21 ± 0.03**	**88.26 ± 0.04**	**0.78 ± 0.00**	**0.92 ± 0.00**	**0.18 ± 0.00**
		90	17.61 ± 0.03	12.42 ± 0.04	8.35 ± 0.03	87.94 ± 0.05	0.77 ± 0.00	0.91 ± 0.00	**0.18 ± 0.00**
		120	17.78 ± 0.10	12.49 ± 0.04	8.39 ± 0.03	87.71 ± 0.13	0.77 ± 0.00	0.91 ± 0.00	0.19 ± 0.00
	60	30	29.25 ± 0.08	21.79 ± 0.08	15.29 ± 0.08	66.61 ± 0.19	0.59 ± 0.00	0.75 ± 0.00	0.32 ± 0.00
		**60**	**28.49 ± 0.03**	**20.74 ± 0.04**	**14.16 ± 0.05**	**68.32 ± 0.07**	**0.63 ± 0.00**	**0.78 ± 0.00**	**0.30 ± 0.00**
		90	28.92 ± 0.09	21.03 ± 0.02	14.29 ± 0.04	67.35 ± 0.20	**0.63 ± 0.00**	0.77 ± 0.00	**0.30 ± 0.00**
		120	29.14 ± 0.12	21.12 ± 0.09	14.32 ± 0.04	66.86 ± 0.27	0.62 ± 0.00	0.77 ± 0.00	0.31 ± 0.00
552	30	**30**	**14.06 ± 0.03**	**8.25 ± 0.11**	**6.48 ± 0.09**	**86.18 ± 0.05**	**0.75 ± 0.00**	**0.92 ± 0.00**	**0.14 ± 0.00**
		60	14.32 ± 0.08	8.91 ± 0.08	7.03 ± 0.06	85.67 ± 0.16	0.73 ± 0.00	0.91 ± 0.00	0.15 ± 0.00
		90	14.47 ± 0.10	9.25 ± 0.09	7.30 ± 0.09	85.36 ± 0.20	0.72 ± 0.00	0.91 ± 0.00	0.15 ± 0.00
		120	14.60 ± 0.08	9.42 ± 0.03	7.44 ± 0.03	85.09 ± 0.16	0.72 ± 0.00	0.91 ± 0.00	0.15 ± 0.00
	60	30	23.83 ± 0.03	**14.57 ± 0.10**	**11.75 ± 0.12**	60.36 ± 0.09	**0.64 ± 0.00**	**0.84 ± 0.00**	**0.22 ± 0.00**
		60	**23.71 ± 0.06**	14.94 ± 0.06	12.07 ± 0.06	**60.78 ± 0.18**	0.63 ± 0.00	**0.84 ± 0.00**	**0.22 ± 0.00**
		90	23.75 ± 0.08	15.44 ± 0.09	12.42 ± 0.06	60.66 ± 0.26	**0.64 ± 0.00**	**0.84 ± 0.00**	0.23 ± 0.00
		120	23.87 ± 0.07	15.50 ± 0.09	12.47 ± 0.08	60.25 ± 0.22	**0.64 ± 0.00**	**0.84 ± 0.00**	0.23 ± 0.00
567	30	30	**22.72 ± 0.04**	**16.47 ± 0.04**	**12.48 ± 0.03**	**84.80 ± 0.05**	**0.64 ± 0.00**	**0.80 ± 0.00**	**0.28 ± 0.00**
		60	22.98 ± 0.07	16.63 ± 0.07	12.93 ± 0.07	84.44 ± 0.10	**0.64 ± 0.00**	**0.80 ± 0.00**	0.29 ± 0.00
		90	23.48 ± 0.18	17.24 ± 0.15	13.48 ± 0.12	83.77 ± 0.25	0.62 ± 0.00	0.79 ± 0.00	0.31 ± 0.00
		120	24.18 ± 0.20	17.98 ± 0.15	14.18 ± 0.12	82.78 ± 0.29	0.61 ± 0.00	0.78 ± 0.00	0.32 ± 0.00
	60	30	**38.38 ± 0.02**	29.51 ± 0.04	**23.24 ± 0.06**	**56.68 ± 0.04**	0.46 ± 0.00	**0.64 ± 0.00**	**0.47 ± 0.00**
		60	39.00 ± 0.07	**29.36 ± 0.01**	23.95 ± 0.01	55.27 ± 0.15	**0.48 ± 0.00**	**0.64 ± 0.00**	0.48 ± 0.00
		90	39.46 ± 0.07	29.96 ± 0.01	24.71 ± 0.03	54.22 ± 0.17	0.46 ± 0.00	0.63 ± 0.00	0.49 ± 0.00
		120	40.39 ± 0.15	30.91 ± 0.08	25.66 ± 0.09	52.01 ± 0.35	0.44 ± 0.00	0.62 ± 0.00	0.51 ± 0.00
584	30	30	23.25 ± 0.08	**16.72 ± 0.06**	**11.00 ± 0.07**	84.88 ± 0.10	0.76 ± 0.00	0.87 ± 0.00	**0.23 ± 0.00**
		60	**22.78 ± 0.04**	16.92 ± 0.04	11.34 ± 0.03	**85.49 ± 0.05**	**0.77 ± 0.00**	0.87 ± 0.00	**0.23 ± 0.00**
		90	22.80 ± 0.02	17.17 ± 0.03	11.51 ± 0.02	85.47 ± 0.03	0.76 ± 0.00	**0.88 ± 0.00**	0.24 ± 0.00
		120	23.30 ± 0.10	17.59 ± 0.10	11.79 ± 0.08	84.82 ± 0.13	0.75 ± 0.00	0.87 ± 0.00	0.25 ± 0.00
	60	30	37.53 ± 0.03	27.65 ± 0.22	**18.33 ± 0.27**	60.48 ± 0.07	0.59 ± 0.00	0.71 ± 0.01	**0.37 ± 0.00**
		60	**35.99 ± 0.05**	**27.29 ± 0.02**	18.40 ± 0.03	**63.67 ± 0.11**	**0.60 ± 0.00**	**0.72 ± 0.00**	**0.37 ± 0.00**
		90	36.04 ± 0.06	27.64 ± 0.06	18.72 ± 0.07	63.56 ± 0.12	0.59 ± 0.00	**0.72 ± 0.00**	0.38 ± 0.00
		120	36.39 ± 0.04	27.83 ± 0.09	18.84 ± 0.12	62.85 ± 0.08	0.58 ± 0.00	0.71 ± 0.00	0.38 ± 0.00
596	30	30	18.66 ± 0.09	13.47 ± 0.11	10.09 ± 0.10	85.82 ± 0.14	0.71 ± 0.00	0.89 ± 0.00	0.21 ± 0.00
		60	**17.87 ± 0.08**	**12.89 ± 0.06**	**9.67 ± 0.03**	**86.99 ± 0.12**	0.74 ± 0.00	0.89 ± 0.00	**0.20 ± 0.00**
		90	17.87 ± 0.09	12.93 ± 0.06	9.71 ± 0.03	86.99 ± 0.13	**0.75 ± 0.00**	0.89 ± 0.00	**0.20 ± 0.00**
		120	17.95 ± 0.05	12.98 ± 0.03	9.76 ± 0.02	86.89 ± 0.07	0.74 ± 0.00	**0.90 ± 0.00**	**0.20 ± 0.00**
	60	30	30.46 ± 0.10	22.78 ± 0.08	17.57 ± 0.08	62.29 ± 0.25	0.52 ± 0.00	0.78 ± 0.00	0.33 ± 0.00
		60	29.00 ± 0.13	21.43 ± 0.14	16.36 ± 0.13	65.83 ± 0.30	0.56 ± 0.00	0.80 ± 0.00	**0.31 ± 0.00**
		90	**28.79 ± 0.05**	**21.35 ± 0.07**	**16.28 ± 0.07**	**66.32 ± 0.13**	**0.57 ± 0.01**	0.80 ± 0.00	**0.31 ± 0.00**
		120	28.83 ± 0.16	21.37 ± 0.16	16.34 ± 0.16	66.22 ± 0.37	**0.57 ± 0.01**	**0.81 ± 0.00**	**0.31 ± 0.00**

Note. Values in bold indicate the best evaluation outcome for each metric in each learning scenario, and grey highlights denote the best model in each scenario based on the best-achieved evaluation metrics. Note. PID: patient identification; PH: prediction horizon; LL: lag length; RMSE: root mean square error; SD: standard deviation; MAE: mean absolute error; MAPE: mean absolute percentage error; r^2^: coefficient of determination; MCC: Matthew’s correlation coefficient; SE: surveillance error; ASE: average surveillance error.

**Table A3:** The evaluation results for non-stacking models created by long short-term memory learners using Ohio 2018 dataset.

PID	PH	LL	Evaluation Metric						
			RMSE ± SD (mg/dL)	MAE ± SD (mg/dL)	MAPE ± SD (%)	r^2^ ± SD (%)	MCC ± SD (%)	SE < 0.5 ± SD (%)	ASE ± SD
559	30	30	**23.12 ± 0.43**	**16.60 ± 0.66**	11.10 ± 0.63	**87.19 ± 0.47**	**0.74 ± 0.01**	0.86 ± 0.01	0.24 ± 0.01
		60	23.51 ± 0.36	16.79 ± 0.54	11.02 ± 0.64	86.76 ± 0.40	**0.74 ± 0.01**	**0.87 ± 0.01**	0.23 ± 0.01
		90	25.50 ± 1.19	17.44 ± 0.64	**10.71 ± 0.13**	84.39 ± 1.44	0.72 ± 0.03	**0.87 ± 0.01**	**0.23 ± 0.00**
		120	32.86 ± 13.20	23.72 ± 10.60	15.55 ± 8.01	71.35 ± 23.13	0.63 ± 0.19	0.78 ± 0.16	0.31 ± 0.15
	60	30	38.39 ± 0.82	27.05 ± 0.53	16.65 ± 0.21	64.46 ± 1.52	0.57 ± 0.01	**0.75 ± 0.00**	0.35 ± 0.00
		60	38.73 ± 4.41	27.75 ± 3.58	17.37 ± 1.50	63.53 ± 8.42	0.54 ± 0.07	0.73 ± 0.05	0.37 ± 0.05
		90	37.77 ± 3.27	26.72 ± 2.04	16.92 ± 0.47	65.46 ± 6.01	0.58 ± 0.02	0.75 ± 0.01	0.35 ± 0.02
		120	**36.08 ± 1.47**	**25.38 ± 0.84**	**16.62 ± 0.25**	**68.60 ± 2.56**	**0.59 ± 0.02**	0.75 ± 0.01	**0.34 ± 0.01**
563	30	30	**21.59 ± 0.64**	**15.33 ± 0.45**	**9.69 ± 0.19**	77.31 ± 1.34	0.72 ± 0.01	**0.89 ± 0.00**	**0.22 ± 0.00**
		60	21.73 ± 0.46	15.52 ± 0.33	9.82 ± 0.32	**77.03 ± 0.96**	**0.73 ± 0.00**	**0.89 ± 0.00**	0.22 ± 0.01
		90	24.91 ± 1.84	17.49 ± 1.38	10.96 ± 1.02	69.71 ± 4.55	0.69 ± 0.03	0.87 ± 0.02	0.24 ± 0.02
		120	24.04 ± 1.89	16.94 ± 1.15	10.65 ± 0.72	71.79 ± 4.43	0.69 ± 0.01	0.87 ± 0.01	0.24 ± 0.01
	60	30	**33.02 ± 0.62**	**24.13 ± 0.61**	**15.07 ± 0.18**	**47.03 ± 2.01**	0.51 ± 0.01	0.75 ± 0.02	**0.33 ± 0.01**
		60	34.44 ± 2.48	25.05 ± 2.24	15.80 ± 1.37	42.17 ± 8.46	0.48 ± 0.09	0.74 ± 0.06	0.35 ± 0.03
		90	34.32 ± 1.23	24.45 ± 1.04	15.16 ± 0.63	42.73 ± 4.13	**0.52 ± 0.01**	**0.77 ± 0.02**	0.34 ± 0.01
		120	34.13 ± 1.59	24.66 ± 1.10	15.27 ± 0.62	43.33 ± 5.27	0.50 ± 0.02	0.76 ± 0.02	0.34 ± 0.01
570	30	30	24.78 ± 3.96	18.97 ± 3.76	8.84 ± 1.30	86.33 ± 4.12	**0.82 ± 0.01**	0.94 ± 0.01	0.16 ± 0.02
		60	25.83 ± 5.11	19.99 ± 4.76	9.28 ± 1.87	85.02 ± 5.59	0.81 ± 0.03	0.93 ± 0.02	0.17 ± 0.03
		90	23.09 ± 2.28	17.15 ± 2.09	8.26 ± 0.74	88.25 ± 2.30	**0.82 ± 0.01**	**0.94 ± 0.00**	**0.15 ± 0.01**
		120	**22.92 ± 1.49**	**16.16 ± 1.15**	**8.04 ± 0.65**	**88.47 ± 1.52**	0.81 ± 0.02	0.94 ± 0.01	**0.15 ± 0.01**
	60	30	38.34 ± 2.65	29.98 ± 2.52	13.56 ± 0.95	67.77 ± 4.48	0.75 ± 0.01	**0.88 ± 0.01**	0.25 ± 0.02
		60	**35.80 ± 1.50**	**26.75 ± 1.85**	**12.68 ± 0.43**	**71.95 ± 2.31**	**0.75 ± 0.00**	**0.88 ± 0.01**	**0.23 ± 0.01**
		90	37.00 ± 2.48	27.94 ± 1.86	13.17 ± 0.99	69.98 ± 4.09	0.75 ± 0.03	0.87 ± 0.02	0.24 ± 0.02
		120	35.80 ± 2.62	25.82 ± 2.70	12.58 ± 0.95	71.89 ± 4.09	0.75 ± 0.02	0.88 ± 0.01	0.23 ± 0.02
575	30	30	**27.20 ± 0.57**	**18.25 ± 0.45**	13.14 ± 0.71	**80.24 ± 0.82**	**0.69 ± 0.00**	0.82 ± 0.02	**0.28 ± 0.01**
		60	27.52 ± 0.76	18.26 ± 0.37	**13.07 ± 0.32**	79.77 ± 1.13	0.69 ± 0.01	**0.82 ± 0.00**	**0.28 ± 0.01**
		90	28.37 ± 0.99	18.89 ± 0.88	13.78 ± 0.69	78.51 ± 1.51	0.68 ± 0.01	0.80 ± 0.01	0.30 ± 0.01
		120	29.33 ± 1.12	19.83 ± 1.63	13.69 ± 0.60	77.03 ± 1.74	0.65 ± 0.05	0.80 ± 0.02	0.29 ± 0.01
	60	30	**38.09 ± 0.03**	**27.47 ± 0.52**	**20.48 ± 1.20**	**61.36 ± 0.07**	**0.54 ± 0.02**	**0.70 ± 0.00**	**0.41 ± 0.01**
		60	39.96 ± 0.84	28.84 ± 0.27	21.39 ± 1.07	57.46 ± 1.78	0.55 ± 0.03	0.68 ± 0.01	0.44 ± 0.01
		90	38.15 ± 0.52	27.58 ± 0.22	20.56 ± 0.49	61.24 ± 1.06	0.52 ± 0.01	0.68 ± 0.01	0.42 ± 0.01
		120	39.47 ± 1.28	28.64 ± 0.43	21.35 ± 0.44	58.48 ± 2.69	0.54 ± 0.01	0.67 ± 0.01	0.43 ± 0.01
588	30	30	**19.23 ± 0.11**	**14.16 ± 0.11**	**8.53 ± 0.12**	**83.77 ± 0.19**	**0.74 ± 0.00**	**0.92 ± 0.00**	**0.19 ± 0.00**
		60	19.60 ± 0.23	14.57 ± 0.15	8.83 ± 0.07	83.13 ± 0.39	0.74 ± 0.01	**0.92 ± 0.00**	**0.19 ± 0.00**
		90	20.33 ± 0.86	15.00 ± 0.73	8.87 ± 0.36	81.84 ± 1.54	0.73 ± 0.01	0.92 ± 0.00	0.19 ± 0.01
		120	21.99 ± 1.74	16.39 ± 1.07	9.64 ± 0.77	78.69 ± 3.39	0.69 ± 0.02	0.91 ± 0.02	0.20 ± 0.02
	60	30	31.32 ± 0.53	23.12 ± 0.56	14.05 ± 0.68	57.00 ± 1.48	0.57 ± 0.01	0.79 ± 0.02	0.30 ± 0.02
		60	**30.46 ± 0.60**	**22.48 ± 0.39**	**14.04 ± 0.23**	**59.33 ± 1.61**	**0.60 ± 0.01**	**0.79 ± 0.01**	**0.30 ± 0.01**
		90	32.01 ± 0.53	23.06 ± 0.33	14.11 ± 0.47	55.07 ± 1.48	0.58 ± 0.02	0.80 ± 0.01	**0.30 ± 0.01**
		120	35.57 ± 4.21	25.60 ± 2.74	15.65 ± 1.69	44.02 ± 13.55	0.50 ± 0.08	0.76 ± 0.03	0.33 ± 0.03
591	30	30	**26.00 ± 0.54**	19.63 ± 0.54	15.81 ± 0.75	**74.78 ± 1.04**	0.58 ± 0.01	0.74 ± 0.00	0.35 ± 0.01
		60	26.33 ± 0.42	**19.55 ± 0.24**	15.65 ± 0.40	74.16 ± 0.83	**0.60 ± 0.00**	**0.75 ± 0.01**	**0.34 ± 0.01**
		90	27.44 ± 1.02	20.46 ± 0.58	**15.63 ± 0.98**	71.90 ± 2.10	0.55 ± 0.05	0.74 ± 0.01	**0.34 ± 0.01**
		120	27.16 ± 0.88	20.13 ± 0.63	15.75 ± 0.85	72.48 ± 1.78	0.57 ± 0.03	0.74 ± 0.02	**0.34 ± 0.01**
	60	30	**36.51 ± 0.20**	**28.36 ± 0.26**	23.32 ± 0.27	**50.32 ± 0.54**	0.37 ± 0.02	**0.63 ± 0.00**	**0.47 ± 0.00**
		60	37.52 ± 0.93	28.36 ± 0.32	**22.47 ± 0.57**	47.52 ± 2.58	0.36 ± 0.04	0.63 ± 0.01	**0.47 ± 0.00**
		90	37.92 ± 1.44	29.32 ± 1.16	24.31 ± 1.51	46.38 ± 4.10	**0.39 ± 0.04**	0.63 ± 0.01	0.48 ± 0.01
		120	37.07 ± 1.67	28.38 ± 1.14	22.37 ± 0.89	48.73 ± 4.57	0.37 ± 0.02	0.63 ± 0.02	0.47 ± 0.02

Note. Values in bold indicate the best evaluation outcome for each metric in each learning scenario, and grey highlights denote the best model in each scenario based on the best-achieved evaluation metrics. Note. PID: patient identification; PH: prediction horizon; LL: lag length; RMSE: root mean square error; SD: standard deviation; MAE: mean absolute error; MAPE: mean absolute percentage error; r^2^: coefficient of determination; MCC: Matthew’s correlation coefficient; SE: surveillance error; ASE: average surveillance error.

**Table A4:** The evaluation results for non-stacking models created by long short-term memory learners using Ohio 2020 dataset.

PID	PH	LL	Evaluation Metric						
			RMSE ± SD (mg/dL)	MAE ± SD (mg/dL)	MAPE ± SD (%)	r^2^ ± SD (%)	MCC ± SD (%)	SE < 0.5 ± SD (%)	ASE ± SD
540	30	30	25.76 ± 1.26	19.38 ± 0.62	14.84 ± 0.24	84.25 ± 1.55	**0.67 ± 0.01**	0.79 ± 0.00	0.31 ± 0.00
		60	**24.84 ± 0.42**	**18.48 ± 0.70**	**13.81 ± 1.24**	**85.37 ± 0.49**	0.67 ± 0.02	**0.80 ± 0.01**	**0.29 ± 0.02**
		90	28.02 ± 3.64	21.40 ± 2.68	15.98 ± 2.30	81.18 ± 4.68	0.63 ± 0.03	0.76 ± 0.03	0.33 ± 0.04
		120	27.92 ± 1.82	21.00 ± 1.99	15.38 ± 2.29	81.48 ± 2.40	0.63 ± 0.02	0.76 ± 0.02	0.32 ± 0.04
	60	30	42.60 ± 1.15	31.84 ± 0.41	23.25 ± 0.53	57.07 ± 2.32	0.48 ± 0.02	0.64 ± 0.01	0.45 ± 0.00
		60	**41.36 ± 0.58**	**30.69 ± 0.37**	**22.40 ± 0.20**	**59.56 ± 1.12**	**0.50 ± 0.02**	**0.66 ± 0.00**	**0.44 ± 0.00**
		90	43.78 ± 2.80	32.44 ± 2.02	23.51 ± 1.66	54.55 ± 5.78	0.50 ± 0.04	0.64 ± 0.02	0.45 ± 0.02
		120	48.17 ± 1.39	34.62 ± 2.09	24.69 ± 2.33	45.10 ± 3.15	0.48 ± 0.04	0.63 ± 0.03	0.48 ± 0.03
544	30	30	21.23 ± 0.53	15.00 ± 0.49	**9.93 ± 0.35**	82.45 ± 0.87	**0.76 ± 0.01**	**0.89 ± 0.00**	**0.21 ± 0.01**
		60	**20.66 ± 0.31**	**14.71 ± 0.43**	9.99 ± 0.53	**83.40 ± 0.50**	0.75 ± 0.01	0.88 ± 0.02	0.22 ± 0.01
		90	22.55 ± 0.45	15.56 ± 0.37	10.40 ± 0.27	80.21 ± 0.79	0.72 ± 0.01	0.88 ± 0.01	0.22 ± 0.00
		120	23.38 ± 2.94	16.49 ± 1.81	11.35 ± 1.30	78.51 ± 5.18	0.71 ± 0.04	0.84 ± 0.03	0.24 ± 0.03
	60	30	31.43 ± 0.05	23.19 ± 0.08	15.59 ± 0.16	61.46 ± 0.12	0.58 ± 0.01	0.76 ± 0.00	0.32 ± 0.00
		60	**30.45 ± 0.12**	**22.09 ± 0.45**	**14.81 ± 0.52**	**63.83 ± 0.29**	**0.59 ± 0.02**	**0.78 ± 0.01**	**0.31 ± 0.01**
		90	32.39 ± 0.61	22.91 ± 0.32	15.40 ± 0.39	59.04 ± 1.55	0.57 ± 0.01	0.76 ± 0.01	0.33 ± 0.01
		120	36.19 ± 1.38	25.61 ± 0.40	17.44 ± 0.10	48.85 ± 3.94	0.52 ± 0.04	0.74 ± 0.01	0.36 ± 0.01
552	30	30	**16.72 ± 0.44**	**10.31 ± 0.24**	**8.04 ± 0.22**	**80.45 ± 1.01**	**0.71 ± 0.02**	**0.90 ± 0.01**	**0.16 ± 0.01**
		60	21.54 ± 3.51	14.67 ± 3.62	11.21 ± 2.37	66.99 ± 10.53	0.59 ± 0.14	0.85 ± 0.04	0.22 ± 0.04
		90	18.81 ± 1.50	12.58 ± 1.52	9.73 ± 0.98	75.16 ± 3.97	0.69 ± 0.01	0.89 ± 0.01	0.19 ± 0.01
		120	20.91 ± 5.44	14.00 ± 4.23	11.01 ± 3.87	68.05 ± 17.09	0.69 ± 0.08	0.85 ± 0.10	0.22 ± 0.08
	60	30	**25.47 ± 0.30**	**16.27 ± 0.24**	**13.02 ± 0.27**	**54.73 ± 1.05**	**0.61 ± 0.01**	**0.83 ± 0.01**	**0.24 ± 0.01**
		60	27.15 ± 1.00	18.20 ± 0.92	15.02 ± 0.93	48.51 ± 3.76	0.58 ± 0.03	0.78 ± 0.02	0.28 ± 0.02
		90	27.51 ± 2.98	17.70 ± 1.96	14.55 ± 1.73	46.78 ± 11.78	0.56 ± 0.06	0.80 ± 0.04	0.27 ± 0.04
		120	40.75 ± 25.37	32.17 ± 26.99	26.17 ± 21.83	45.82 ± 170.04	0.33 ± 0.44	0.60 ± 0.38	0.53 ± 0.49
567	30	30	26.21 ± 1.00	18.74 ± 1.00	14.41 ± 1.01	79.74 ± 1.56	**0.61 ± 0.01**	0.77 ± 0.01	0.32 ± 0.02
		60	25.54 ± 0.32	18.38 ± 0.28	13.83 ± 0.55	80.78 ± 0.48	**0.61 ± 0.01**	**0.78 ± 0.00**	**0.31 ± 0.01**
		90	**24.64 ± 0.97**	**17.85 ± 0.81**	**13.48 ± 0.66**	**82.10 ± 1.41**	0.60 ± 0.01	0.78 ± 0.01	**0.31 ± 0.01**
		120	27.89 ± 3.45	20.96 ± 3.26	16.17 ± 2.94	76.86 ± 5.47	0.57 ± 0.05	0.74 ± 0.04	0.35 ± 0.06
	60	30	43.16 ± 1.27	32.69 ± 1.21	27.34 ± 1.23	45.19 ± 3.24	0.44 ± 0.02	0.60 ± 0.02	0.53 ± 0.02
		60	**40.13 ± 1.22**	**30.57 ± 1.14**	**25.05 ± 1.96**	**52.61 ± 2.86**	**0.45 ± 0.01**	**0.62 ± 0.02**	**0.50 ± 0.03**
		90	42.89 ± 2.29	32.84 ± 2.03	26.97 ± 2.57	45.79 ± 5.74	0.41 ± 0.01	0.60 ± 0.02	0.53 ± 0.03
		120	45.08 ± 4.52	34.30 ± 3.01	26.78 ± 0.56	39.83 ± 12.30	0.40 ± 0.06	0.58 ± 0.04	0.54 ± 0.04
584	30	30	26.87 ± 0.77	19.56 ± 0.72	13.10 ± 0.55	79.81 ± 1.16	0.72 ± 0.02	0.84 ± 0.01	0.26 ± 0.01
		60	**25.31 ± 1.32**	**18.27 ± 0.95**	**11.49 ± 0.52**	**82.05 ± 1.89**	**0.75 ± 0.01**	**0.86 ± 0.01**	**0.23 ± 0.01**
		90	25.93 ± 1.03	19.25 ± 0.82	13.00 ± 0.65	81.19 ± 1.47	0.74 ± 0.01	0.85 ± 0.01	0.26 ± 0.01
		120	27.62 ± 0.80	20.65 ± 1.21	13.36 ± 0.35	78.66 ± 1.24	0.72 ± 0.02	0.84 ± 0.01	0.27 ± 0.00
	60	30	**41.45 ± 1.58**	**31.50 ± 1.91**	**21.43 ± 2.17**	**51.75 ± 3.64**	0.55 ± 0.03	**0.67 ± 0.04**	**0.42 ± 0.04**
		60	42.14 ± 1.60	32.72 ± 1.78	23.12 ± 1.60	50.12 ± 3.74	0.55 ± 0.01	0.64 ± 0.04	0.45 ± 0.03
		90	41.75 ± 0.90	32.60 ± 0.83	22.86 ± 1.00	51.08 ± 2.11	**0.56 ± 0.01**	0.65 ± 0.02	0.44 ± 0.02
		120	47.83 ± 3.54	37.15 ± 4.34	25.97 ± 4.37	35.58 ± 9.66	0.46 ± 0.05	0.59 ± 0.07	0.50 ± 0.08
596	30	30	**19.96 ± 0.28**	**14.31 ± 0.03**	**10.83 ± 0.18**	**83.78 ± 0.45**	**0.70 ± 0.01**	**0.87 ± 0.00**	**0.23 ± 0.00**
		60	21.15 ± 0.65	15.31 ± 0.40	11.64 ± 0.41	81.77 ± 1.12	0.69 ± 0.01	0.86 ± 0.01	0.24 ± 0.01
		90	22.54 ± 0.82	16.38 ± 0.95	12.32 ± 0.90	79.29 ± 1.50	0.66 ± 0.04	0.85 ± 0.01	0.25 ± 0.01
		120	33.46 ± 10.29	25.29 ± 8.45	19.64 ± 6.92	51.54 ± 25.67	0.50 ± 0.16	0.75 ± 0.10	0.36 ± 0.11
	60	30	30.97 ± 0.19	22.79 ± 0.17	17.23 ± 0.22	61.02 ± 0.48	0.52 ± 0.01	0.78 ± 0.00	0.33 ± 0.00
		60	**30.28 ± 0.72**	**22.17 ± 0.71**	**16.97 ± 0.45**	**62.72 ± 1.77**	**0.56 ± 0.02**	**0.79 ± 0.00**	**0.32 ± 0.01**
		90	31.70 ± 1.25	23.44 ± 1.22	17.94 ± 1.21	59.12 ± 3.24	0.52 ± 0.03	0.78 ± 0.01	0.34 ± 0.02
		120	36.31 ± 9.68	27.21 ± 8.48	21.03 ± 6.87	43.87 ± 30.66	0.43 ± 0.21	0.71 ± 0.13	0.40 ± 0.11

Note. Values in bold indicate the best evaluation outcome for each metric in each learning scenario, and grey highlights denote the best model in each scenario based on the best-achieved evaluation metrics. Note. PID: patient identification; PH: prediction horizon; LL: lag length; RMSE: root mean square error; SD: standard deviation; MAE: mean absolute error; MAPE: mean absolute percentage error; r^2^: coefficient of determination; MCC: Matthew’s correlation coefficient; SE: surveillance error; ASE: average surveillance error.
